# Development and pilot testing of PROACTIVE: A pediatric onco‐critical care capacity and quality assessment tool for resource‐limited settings

**DOI:** 10.1002/cam4.5395

**Published:** 2022-11-02

**Authors:** Anita V. Arias, Firas M. Sakaan, Maria Puerto‐Torres, Zebin Al Zebin, Parthasarathi Bhattacharyya, Adolfo Cardenas, Sanjeeva Gunasekera, Joyce Kambugu, Kirill Kirgizov, Jaime Libes, Angelica Martinez, Nune V. Matinyan, Alejandra Mendez, Janet Middlekauff, Katie R. Nielsen, Andrew Pappas, Hong Ren, Rana Sharara‐Chami, Silvio F. Torres, Jennifer McArthur, Asya Agulnik

**Affiliations:** ^1^ Division of Critical Care St. Jude Children's Research Hospital Memphis Tennessee USA; ^2^ Department of Global Pediatric Medicine St. Jude Children's Research Hospital Memphis Tennessee USA; ^3^ Pediatric Hematology and Oncology King Hussein Cancer Center Amman Jordan; ^4^ Department of Pediatric Oncology Critical Care Tata Medical Center Kolkata India; ^5^ Department of Pediatric Oncology National Cancer Institute Sri Lanka Maharagama Sri Lanka; ^6^ Department of Pediatric Oncology Uganda Cancer Institute Kampala Uganda; ^7^ St. Jude Global Memphis Tennessee USA; ^8^ Department of Pediatric Hematology and Oncology University of Illinois College of Medicine Peoria Illinois USA; ^9^ Pediatric Hemato‐Oncology Unit Hospital General de Tijuana Tijuana Baja California Mexico; ^10^ Pediatric Critical Care Unidad Nacional de Oncología Pediátrica (UNOP) Guatemala City Guatemala; ^11^ Division of Pediatric Critical Care University of Washington Seattle Washington USA; ^12^ Department of Global Health University of Washington Seattle Washington USA; ^13^ Department of Pediatric Intensive Care Unit Shanghai Children's Medical Center Shanghai China; ^14^ Department of Pediatric and Adolescent Medicine American University of Beirut Medical Center Beirut Lebanon; ^15^ Pediatric Intensive Care Unit Hospital Universitario Austral Buenos Aires Argentina

**Keywords:** critical care medicine, global health, health quality of care, pediatric cancer, quality improvement

## Abstract

**Background:**

Nearly 90% children with cancer reside in low‐ and middle‐income countries, which face multiple challenges delivering high‐quality pediatric onco‐critical care (POCC). We recently identified POCC quality and capacity indicators for PROACTIVE (PediatRic Oncology cApaCity assessment Tool for IntensiVe carE), a tool that evaluates strengths and limitations in POCC services. This study describes pilot testing of PROACTIVE, development of center‐specific reports, and identification of common POCC challenges.

**Methods:**

The original 119 consensus‐derived PROACTIVE indicators were converted into 182 questions divided between 2 electronic surveys for intensivists and oncologists managing critically ill pediatric cancer patients. Alpha‐testing was conducted to confirm face‐validity with four pediatric intensivists. Eleven centers representing diverse geographic regions, income levels, and POCC services conducted beta‐testing to evaluate usability, feasibility, and applicability of PROACTIVE. Centers' responses were scored and indicators with mean scores ≤75% in availability/performance were classified as common POCC challenges.

**Results:**

Alpha‐testing ensured face‐validity and beta‐testing demonstrated feasibility and usability of PROACTIVE (October 2020–June 2021). Twenty‐two surveys (response rate 99.4%) were used to develop center‐specific reports. Adjustments to PROACTIVE were made based on focus group feedback and surveys, resulting in 200 questions. Aggregated data across centers identified common POCC challenges: (1) lack of pediatric intensivists, (2) absence of abstinence and withdrawal symptoms monitoring, (3) shortage of supportive care resources, and (4) limited POCC training for physicians and nurses.

**Conclusions:**

PROACTIVE is a feasible and contextually appropriate tool to help clinicians and organizations identify challenges in POCC services across a wide range of resource‐levels. Widespread use of PROACTIVE can help prioritize and develop tailored interventions to strengthen POCC services and outcomes globally.

## Graphical abstract

We described the development and pilot testing of PROACTIVE, a tool to assess pediatric onco‐critical care services in hospitals across a wide range of resource‐levels. Collected data was used to create center‐specific reports and identify common challenges, which included lack of adequate pediatric intensivists, training programs, and supportive care resources.

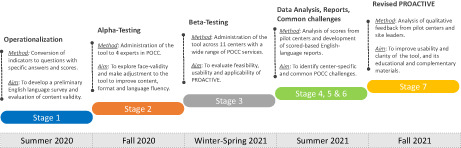

## 1 INTRODUCTION

Globally, an estimated 400,000 new cases of childhood cancer are diagnosed annually with nearly 90% occurring in low‐ and middle‐income countries (LMICs).^1^, ^2^, ^3^ Hospitalized pediatric cancer patients are at high risk for clinical deterioration with approximately 40% requiring admission to the pediatric intensive care unit (PICU) at least once during cancer‐directed therapy.^4^, ^5^, ^6^ Moreover, mortality for these patients is around 28% in high‐income countries^4^ and significantly higher in LMICs due to multiple challenges delivering high‐quality pediatric onco‐critical care (POCC)^7^, ^8^, ^9^, ^10^ or the ability to identify, monitor, or treat pediatric oncology patients who develop critical illness independent of their physical hospital location or the presence of a PICU.

Reducing disparities in pediatric cancer outcomes is a global imperative. Recently, the World Health Organization (WHO) established the Global Initiative for Childhood Cancer with the goal to achieve at least 60% survival for all children with cancer by 2030.^11^, ^12^ This initiative has led to an increase interest to identify areas of opportunity to improve pediatric oncology services.

The use of capacity and quality metrics to evaluate and benchmark systems has been integrated into healthcare programs since the 1990s.^13^ Since then, multiple studies have demonstrated that improving processes of care, ICU structure, and using quality improvement methodologies improve patient outcomes and reduce costs.^14^ While capacity and quality indicators aid healthcare teams to identify weakness and map quality of care, benchmarking is an effective method to measure and analyze internal and external performances against other providers and institutions, enabling improvement efforts in healthcare delivery.^15^, ^16^ However, few tools exist to measure healthcare performance in LMICs and a lack of indicators to measure capacity and quality of care in POCC.

Recently, our team used an expert consensus process to identify POCC capacity and quality indicators to create PROACTIVE (PediatRic Oncology cApaCity assessment Tool for IntensiVe carE)^17^; a tool to evaluate POCC services in resource‐limited hospitals, defined as hospital with a broad range of self‐identified resource limitations including personnel, medications, equipment, and material supplies^18^ needed to treat children with cancer.

The objectives of this study were: (1) Operationalize PROACTIVE via an electronic tool using identified POCC indicators and pilot test across a range of hospitals providing pediatric hematology‐oncology (PHO) care to assess its usability, feasibility, and applicability; (2) develop a scored‐based report to summarize PROACTIVE results to help centers prioritize among improvement opportunities; and (3) identify an initial set of common challenges and improvement opportunities for the delivery and provision of POCC services globally.

## 2 METHODS

**FIGURE 1:**
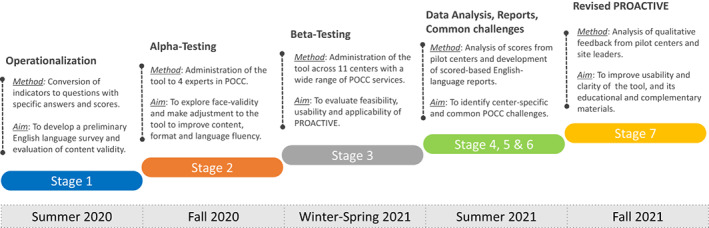
Stages in the development and pilot testing of PROACTIVE. The diagram details the process in the development and validation of the PROACTIVE tool in 7 stages: 1) Operationalization of Indicators, 2) Alpha‐testing, 3) Beta‐testing, 4) Data Analysis, 5) Development of PROACTIVE reports, 6) Identification of Common POCC challenges, and 7) Revised PROACTIVE tool.

This study was approved by the St. Jude Children's Research Hospital Institution Review Board as an exempt quality improvement project. Development and pilot testing of PROACTIVE was performed in stages as shown in Figure 1 and described below:

### 2.1 Stage 1—Operationalization of Indicators

The original 119 PROACTIVE indicators previously developed through expert consensus^17^ and 17 oncology indicators from PrOFILE (Pediatric Oncology Facility Integrated Local Evaluation tool)^19^ were categorized within a framework of 8 domains and 22 subdomains (eTable S1).

These indicators were converted into questions; for example, the indicator “trained nursing staff in pediatric critical care” in the Core Team subdomain was operationalized as: “Is nursing staff formally trained in pediatric critical care part of the primary medical team responsible for the care of critically ill PHO patients?”. Composite indicators were divided into individual components; for example, “access to blood products” was split into platelets and packed red blood cells.

Questions were iteratively adjusted for syntax and content validity and were divided into two English‐language surveys. The *PICU Survey* contained questions assessing POCC services in hospital areas designated for the care of critically ill PHO patients and was answered by a pediatric intensivist or a physician managing critically ill patients. The *Oncology Survey* contained items assessing capacity and quality of care provided to PHO patients on the wards and was answered by a pediatric oncologist. Both electronic surveys were administered using the Research Electronic Data Capture (REDCap) software.^20^

Survey response choices included dichotomous, numerical, and a five‐point Likert scale. To reduce ambiguity in the Likert‐scale responses, answers were assigned equally distanced percentages between choices (eTable S2). Furthermore, all answers were assigned a numerical score from 0 to 5, where 5 indicated performance and/or availability of an item 100% of the time and 0 indicated poor performance and/or absence of an item, allowing for quantitative data analysis and comparison among centers.

### 2.2 Stage 2—Alpha‐testing

To explore face‐validity, PROACTIVE was alpha‐tested (Fall 2020) by four pediatric intensivists with extensive experience in POCC from three different hospitals in the United States and Mexico. Participants were selected from the panel of experts who contributed to the initial PROACTIVE study^17^ and evaluated the consensus derived onco‐critical care components of the survey. Alpha‐testers evaluated the content, format, and language of the tool, ensured answers choices had appropriate scores, questions reflected the original indicators, and were correctly assigned to the PICU and Oncology surveys.

### 2.3 Stage 3—Beta‐testing

Beta‐testing occurred between January and June 2021 with 11‐centers across 11‐countries. Testing sites were selected from hospitals managing a high volume of PHO patients and collaborating with St. Jude Global.^21^ Sites were chosen to represent diverse geographic regions, country economic levels based on the World Bank classification (2021 fiscal year),^22^ POCC services, and hospital organizations ranging from general hospitals with no PICUs to specialized pediatric cancer centers (Table 1 and eFigure S1). In addition, these centers managed a high volume of pediatric oncology patients defined as >20% of all hospitalizations due to cancer‐related reasons or being a pediatric cancer referral center for their region. PROACTIVE was delivered in three phases (preparation, assessment, and interpretation/action), each lasting 4–12 weeks (eFigure S2). This was carried out through virtual educational prerecorded lectures and monthly live mentoring sessions focusing on the steps for each phase. Systematic feedback was requested from participating teams to improve PROACTIVE and supportive materials (see below).

**TABLE 1:** Characteristics of hospitals participating in PROACTIVE Beta‐Testing (*n* = 11)

Characteristic	No. of centers (%)
World bank regions	
East Asia and Pacific	1 (9.1%)
Europe and Central Asia	1 (9.1%)
Latin America and the Caribbean	3 (27.2%)
Middle East and North Africa	2 (18.2%)
North America	1 (9.1%)
South Asia	2 (18.2%)
Sub‐Saharan Africa	1 (9.1%)
Income level^a^	
High‐income countries	1 (9%)
Upper‐Middle income countries	7 (64%)
Low‐Middle income countries	2 (18%)
Low‐income countries	1 (9%)
Type of hospital	
Pediatric Cancer Hospital	2 (18%)
Adult + Pediatric Cancer Center	4 (37%)
Children's Hospital	2 (18%)
General Adult + Pediatric Hospital	3 (27%)
ICUs	
PICU alone	3 (27%)
PICU + IMCU/HDU	6 (55%)
IMCU/ HDU alone	1 (9%)
No ICU/IMCU/HDU	1 (9%)
Bone marrow transplant	
Transplant Unit	6 (55%)
No Transplant Unit	5 (45%)
Staffing	
Pediatric Intensivists	8 (73%)
Trained pediatric critical care nurses	7 (64%)

Abbreviations: HDU, high‐dependency unit; IMCU, intermediate care unit; PICU, pediatric intensive care unit.

^a^ Based on the World Bank classification (2021 fiscal year)^22^: we classified as HIC those with a gross national income per capital (GNI) of ≥ US$12,695, as UMIC those with a GNI of US$4096‐US$12,695, as LMIC those with a gross national income per capita (GNI) of US$1046–4095, and as LIC those with a GNI ≤ US$1046.

During the *preparation phase*, each beta‐testing site identified a team leader responsible for recruiting additional collaborators (site core team) and verifying collected data. The site core teams consisted of physicians and nurses who routinely care for critically ill PHO patients and could answer questions about their unit structure and resources.

During the *assessment phase*, each core team collected the following data:

- *Quantitative data*: Included survey questions collecting objective data regarding the center's structure, processes, and outcomes (e.g., PHO admissions), and subjective data on providers' perceptions about the quality of services offered to critically ill PHO patients (e.g., rating their service capacity).
- *Qualitative data*: Obtained from team leaders who completed 2 English‐language electronic surveys via Qualtrics Research Core^23^ to provide feedback on content and clarity of PROACTIVE questions, usability of online platform (REDCap), training process, and supportive materials (eTable S3). Leaders also participated in three focus group meetings conducted in English via WebEx, a secure online meeting platform, to discuss challenges in completing each phase of PROACTIVE. This helped identify confusing or difficult questions to improve clarity and assess appropriateness of response options.

During the *interpretation and action phase*, PROACTIVE reports were provided to each pilot center summarizing collected data. See Stage 5 for PROACTIVE report development.

### 2.4 Stage 4—Data analysis

Questions were scored based on responses from 0 to 5. Missing or unknown data were assigned a score of zero to identify areas requiring improvement and encourage better record keeping.^16^ Additionally, some questions considered inadequate to assess POCC services by themselves were still included as balancing measures,^16^ but were not scored or included in the aggregate data.

Individual meetings with each core team were performed by two authors (AVA, FS) to check data consistency and discuss outliers and reasons for disagreement among the 37 questions included in both surveys. This helped identify proper assignment of overlapping questions and corroborate scores used for data analysis. An additive aggregation technique was used to provide a total score to each domain and subdomain, and their final percentage score was calculated using the total points in each domain and subdomain divided by the maximum achievable points. Data were then used to develop the PROACTIVE report.

The qualitative data collected from team leaders via electronic surveys and WebEx meetings were analyzed by two authors (AVA, FS) and used to adjust the PROACTIVE tool and online educational and supportive materials.

### 2.5 Stage 5—PROACTIVE center reports

After data collection, each center received a PROACTIVE report. These reports were developed and structured based on PrOFILE^24^, ^25^ and the Clinical Sustainability Assessment Tool (CSAT)^26^ reports to help hospitals interpret their PROACTIVE results and prioritize among multiple improvement opportunities. To confirm accuracy, the team leaders reviewed their report graphs and domain/subdomain scores for major inconsistencies or unexpected results. The team leaders also participated in two focus group meetings to provide feedback on the report's clarity, user‐friendliness, and format.

### 2.6 Stage 6—Common Challenges in POCC

Aggregated data for the 11 beta‐testing sites were used to identify common challenges in POCC defined as any indicator obtaining a mean score ≤75% in availability and/or performance across all centers.

### 2.7 Stage 7—Revised PROACTIVE tool

Improvements to PROACTIVE were made based on the qualitative data collected by two authors (AVA, FS) and feedback gathered from the core teams and leaders. Changes were made to the tool to improve its usability and clarity, including improvement of wording, concepts, and definitions.

## 3 RESULTS

### 3.1 Stages 1 and 2 (Operationalization and Alpha‐testing)

**FIGURE 2:**
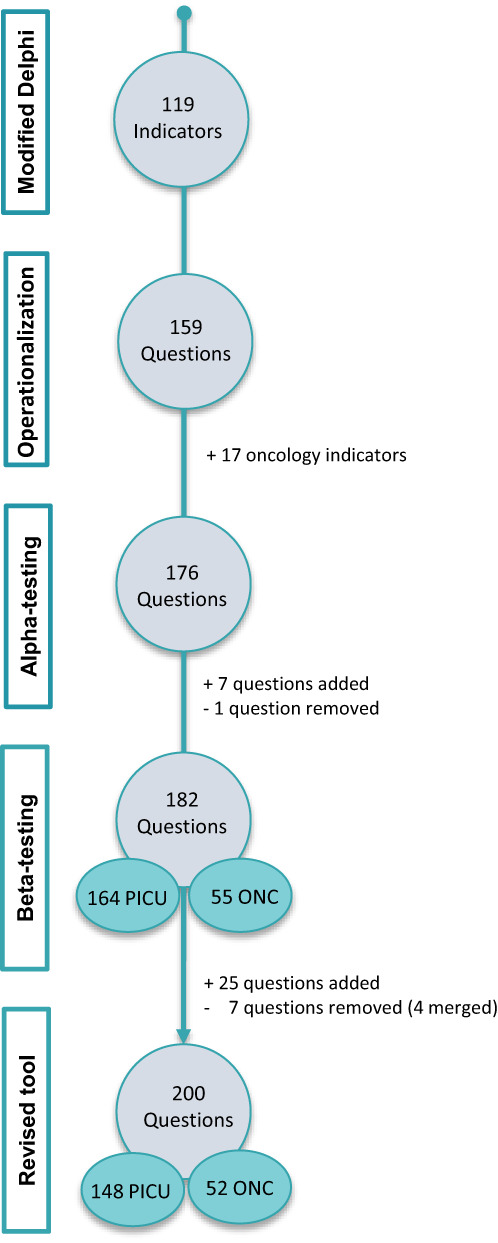
Number of questions per stage. An initial set of 119 consensus‐derived quality and capacity POCC indicators^17^ were operationalized into 159 questions; these questions plus 17 oncology questions^19^ were subjected to alpha‐testing (176 questions). A total of 182 questions were then included for beta‐testing (164 PICU and 55 oncology questions, with 37 questions included in both surveys), resulting in a final PROACTIVE tool containing 200 questions (148 PICU and 52 oncology questions) to assess POCC services.

The original 119 capacity and quality indicators were operationalized into 159 questions; 17 oncology questions from PrOFILE (eTable S4) were also included, resulting in 176 questions used in alpha‐testing (Figure 2). Based on alpha‐testing recommendations, revisions to PROACTIVE included reduction of one semantically equivalent question and further dividing composite indicators into seven new items. Several questions were rephrased for clarity. Additionally, alpha‐testers examined the distribution of questions between the 2 surveys (PICU and Oncology); 37 items received conflicting recommendations and were included in both surveys for evaluation during beta‐testing. This process resulted in a pilot PROACTIVE tool containing 182 questions divided between 2 surveys, 164 questions in the PICU survey, and 55 questions in the Oncology survey (eTable S5). Surveys were also standardized in format, instructions, and answer choices.

### 3.2 Stage 3 (Beta‐Testing)

All beta‐testing sites allocated at least 2 h per week to the process of data collection and discussion of answers. The PROACTIVE tool took approximately 12 h over 4 to 6 weeks to complete (average of 4.2 weeks), with site leaders reporting this time to be adequate for data collection.

Core teams varied in size (2–6 members) and a total of 38 multidisciplinary healthcare providers participated in data collection. Twenty‐two healthcare providers (2 per center) representing the PICU and oncology team (See eTable S6 for respondents' demographics) were responsible for entering collected data into REDCap. Centers reported an age limit for pediatric services ranging from 16 to 19 years old and a median of 1200 PHO hospital admissions (range 80–7041) in the prior 12 months.

### 3.3 Stage 4 (Data Analysis)

Completion rate for both surveys was 99.4%; a total of 22 surveys were submitted with minimal missing data; only 13 items were left unanswered due to inability to retrieve data for these questions by some centers. Missing data were mostly found in the National context and Outcomes domains due to limited physician knowledge about national policies and lack of institutional registries collecting patient outcomes.

Discordance in the answers for the 37 overlapping questions between the PICU and Oncology surveys varied by beta‐testing site and ranged from 0 to 11 questions (0% to 29.7%). These were then reviewed with each center to clarify problems with the question items and identify their proper assignment to either the PICU or oncology survey.

The qualitative feedback data were synthesized through iterative review and suggestions were used to guide revisions to the PROACTIVE tool and supportive materials (See stage 7, revised PROACTIVE tool).

### 3.4 Stage 5 (Report development)

Center‐specific PROACTIVE reports provided a score‐based and descriptive analysis of results across all domains and subdomains. Based on recommendations, adjustments to improve visualization of the reports included a combined total score in bar‐graph format for each domain and subdomain including results from both surveys, and a separate area highlighting the top 5 highest‐ and lowest‐scored subdomains for easier interpretation of strengths and weaknesses (eFigure S3). Team leaders described the final PROACTIVE report as useful to visualize data and identify areas of opportunity for improvement in POCC services in their centers. In addition, the PROACTIVE report was used by some centers to advocate for additional resources to improve their intensive care and POCC capacity. One team used their PROACTIVE report to request echocardiography 24 hours per day, 7 days a week, resulting in the formation of a 24‐hour on‐call echocardiography group. Another team used their report to advocate for nursing education resulting in the development of a POCC workshop for nurses. A third center used their report to justify starting a blood bank program to address shortages in blood products.

### 3.5 Stage 6 (Common Challenges)

The most common POCC challenges or the indicators with the lowest mean scores in availability and/or performance across the 11 centers (Table 2) included: (1) lack of pediatric intensivist resulting in inability to provide coverage 24 hours per day, 7 days a week, (2) absence of abstinence and withdrawal symptoms monitoring, (3) shortage of supportive care resources such as blood products in less than 1 hour and (4) access to intracranial pressure (ICP) monitoring devices, and (5) lack of training programs for physicians and nurses in critical care.

**TABLE 2:** Common challenges in POCC services identified by the PROACTIVE assessment

Domains	Indicators	Availability/Performance (%/N)	
		<75%^a^	<50%^b^
National context	Natl. pediatric critical care nursing training programs		45% (5/11)
	Natl. public funded healthcare program covering most cost		36% (4/11)
Facility & local context	Presence of a pediatric critical care fellowship program		45% (5/11)
Personnel	A pediatric intensivist part of the core team	73% (8/11)	
	A PHO physician part of core team	73% (8/11)	
	Trained pediatric critical care nurses part of the core team	64% (7/11)	
	Availability of pediatric neurosurgeon consultants		36% (4/11)
	In‐House pediatric intensivists 24 h/7 days a week		27% (3/11)
Service capacity	Transfer of deteriorating patients to the PICU in <4 h	73% (8/11)	
	Transfer of deteriorating patients to the PICU in <1 h		45% (5/11)
	Availability of shoe covers		45% (5/11)
Service integration	Daily multidisciplinary rounds led by a pediatric intensivist	73% (8/11)	
	Tracking of unplanned extubations	55% (6/11)	
	System to monitor sedation and/or delirium	55% (6/11)	
	System to monitor abstinence and withdrawal		27% (3/11)
Supportive services	Inadequate nursing staff *almost never* affecting patient care	73% (8/11)	
	System to monitor, prevent, and address medication shortage		45% (5/11)
	Availability of ultrasound studies within 24 h		36% (4/11)
	Availability of emergency blood products in <1 h		27% (3/11)
Meds & equipment	Renal replacement therapy	55% (6/11)	
	Availability of ICP monitors		27% (3/11)
Outcomes	A patient data registry for PHO/BMT patients	73% (8/11)	
	Collaboration to compare or benchmark outcomes	64% (7/11)	

Abbreviations: BMT, bone marrow transplant; ICP, intracranial pressure; IMCU, intermediate medical care unit; IV, intravenous; PHO, pediatric hematology‐oncology patient; PICU, pediatric intensive care unit.

^a^ In green: PROACTIVE indicators obtaining a mean score <75% in availability and/or performance across all centers.

^b^ In yellow: PROACTIVE indicators obtaining a mean score <50% in availability and/or performance across all centers.

**FIGURE 3:**
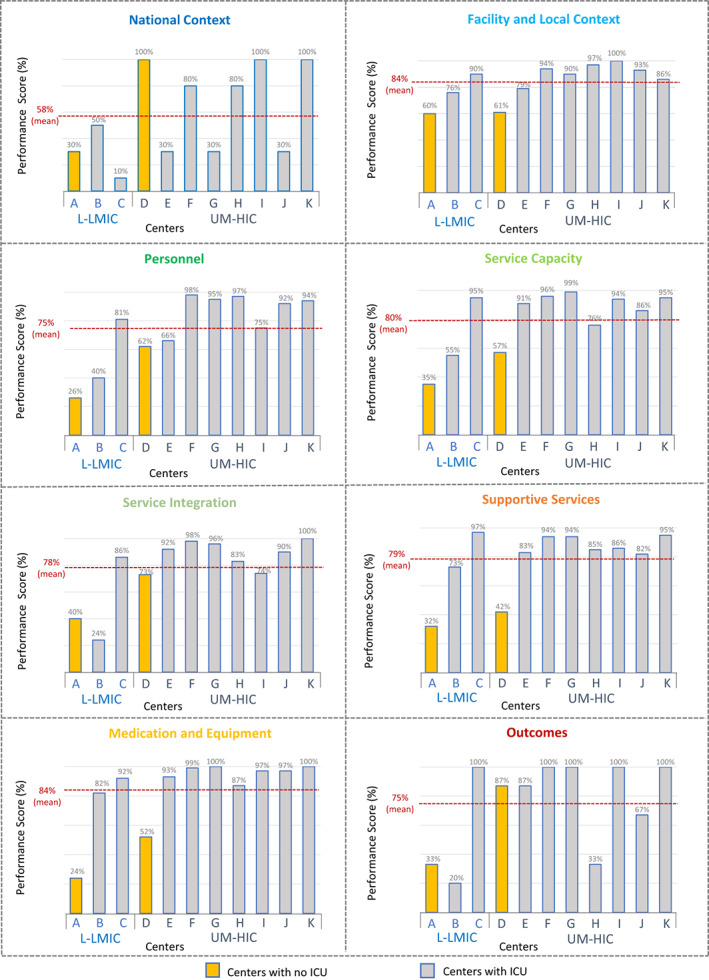
Aggregated data per domain. The centers' average performance per domain is represented in this figure. Centers are ranked by country income level, from low‐ (A) to high‐income level (K), with 2 centers (yellow) not having a designated PICU area. Overall, centers with no PICUs scored inferiorly in most domains independently of their country income level. The National Context obtained the lowest mean score (58%) across the 11 centers, while the Facility/Local context and the Medication/Equipment domains received the highest scores (84%). Abbreviations: L‐LMIC, Low‐ and Low‐Middle Income Countries; UM‐HIC, Upper‐Middle and High‐Income Countries; ICU, Intensive Care Unit.

Additionally, aggregated PROACTIVE data comparing centers across domains revealed variability in POCC services in different resource settings. In general, centers with no designated PICU areas (2/11 centers) scored lower in most domains regardless of their country‐income level, with both centers having the lowest scores in the Supportive Services and Medication/Equipment domains (Figure 3). Across all centers, the National Context domain obtained the lowest mean score (58%), and the Facility/Local Context and Medication/Equipment domains had the highest scores, both with a mean score of 84% (Figure 3).

### 3.6 Stage 7 (Revised PROACTIVE tool)

Based on feedback, 25 questions were added to subdomains considered too narrow to capture all aspects of composite indicators, including question to further assess training, palliative care services, protocols, outcomes, and impact of COVID‐19 pandemic on staffing (eTable S7). Seven questions were removed, three were not part of the original PROACTIVE indicators^17^ and were deemed irrelevant. The remaining four questions were merged with other similar questions. Thirty‐four questions were rephrased to improve clarity.

Additionally, 37 questions initially assigned to both surveys were split between the PICU and Oncology surveys. This resulted in a final set of 200 questions among 8 domains and 22 subdomains divided into 2 surveys with 148 questions assigned to the PICU and 52 questions to the Oncology survey (eTable S8). The Likert scale was also adjusted to a seven‐point scale based on feedback and literature findings indicating that a seven‐point scale is more reliable and useful for scales without a strong floor/ceiling effect,^27^ and more appropriate for electronically‐distributed unsupervised surveys.^28^ Furthermore, the online educational and supportive materials were adjusted to improve content, wording, layout, and length of the presentations for each PROACTIVE phase to better support future cohorts.

## 4 DISCUSSION

Lack of indicators or pragmatic assessment tools prevent hospitals from evaluating and benchmarking their POCC services, which limit their ability to systematically improve delivered care. PROACTIVE is the first diagnostic tool to assess capacity and quality of care in POCC services among hospitals with variable resources. Alpha‐testing ensured face‐validity, and beta‐testing confirmed usability, feasibility, and global applicability of the tool. Beta‐testing was also used to generate center‐specific reports to summarize PROACTIVE results and help centers prioritize improvement opportunities, advocate for resources, and improve POCC services. Common POCC challenges identified by the PROACTIVE pilot allows for some preliminary recommendations to improve care of critically ill PHO patients globally.

Prior studies have shown that patients living in regions with more healthcare resources do not always receive higher quality care or have better clinical outcomes than those living in resource‐limited regions.^29^, ^30^, ^31^ Beta‐testing PROACTIVE sites described limited access to multiple supplies needed for POCC (e.g., ICP monitoring devices), however, whether greater availability of these resources would result in better patient outcomes remains uncertain. PROACTIVE, like other essential medication or equipment lists,^32^, ^33^ can be used by centers to prioritize among multiple competing supplies and materials requests. Anecdotally, some centers in our study successfully used the PROACTIVE report to advocate for additional resources to their institutions to improve local POCC capacity.

Similarly, although only two participating centers in our study had no formal PICUs, these centers reported the most difficulty providing high‐quality POCC. Despite evidence supporting that having a dedicated PICU reduces morbidity and mortality,^34^, ^35^ optimizes staffing efficiency, and facilitates development of expertise among physicians and nurses,^36^ PICUs are not universally available. Even if available, PICUs often face limited bed capacity or staff availability, and their presence does not guarantee access for critically ill patients deemed high risk of mortality, or the consistent delivery of high‐quality POCC. More data are needed on how availability of PICU resources influences quality of care and clinical outcomes for POCC patients.

Notably, the PROACTIVE pilot occurred during the COVID‐19 pandemic, with many sites reporting increased strain in available resources. An important challenge reported by beta‐testing sites was the lack of consistent access to blood products, a finding also reported in many LMICs due to a decrease in donors.^37^ Furthermore, access to life‐saving interventions and critical care services have been disproportionally affected in LMICs resulting in poor survival.^37^ The true magnitude of the impact the pandemic on the care of critically ill pediatric cancer patients is likely more broad and requires further study.

Like other studies, we found that there is a global need for more pediatric critical care and POCC training for physicians and nurses.^36^, ^38^, ^39^, ^40^ Several programs are addressing this imperative, including the St. Jude Global Academy in POCC,^41^ the St. Jude annual POCC symposium,^42^ and the virtual simulation on critical care complications of SCT/cellular therapies by MD Anderson Cancer Center.^43^ The online St. Jude Global Academy in POCC,^41^ for example, covers relevant topics for the multidisciplinary teams caring for critically ill PHO patients in resource‐limited settings and describes best practices in POCC. Besides the St. Jude Global resources, the WHO Global Initiative for Childhood Cancer (GICC) has developed several tools and trainings to support capacity building in childhood cancer globally. These include the CureAll implementation module,^44^ specific regional courses in early cancer diagnosis,^45^ and training in POCC. We hope PROACTIVE can help advance the WHO GICC mission by assessing baseline POCC services within hospital and highlighting urgent priorities that align with country‐specific and regional goals.

In addition to education, there is an urgent need to improve access to free scientific literature by making more articles open access to promote equity and allow clinicians in LMICs to learn from new knowledge in this field.^46^ Likewise, use of validated tools to monitoring iatrogenic withdrawal symptoms such as the Withdrawal Assessment Tool (WAT‐1)^47^ and Sophia Observation withdrawal Symptoms‐scale (SOS)^48^ were rarely used at our pilot‐sites, a finding also reported in a recent meta‐analysis.^49^ To improve systematic monitoring of iatrogenic withdrawal symptoms in patients receiving prolonged analgesia and sedation, the multidisciplinary teams should be trained to use these tools and encouraged to study their impact in their settings. Available tools should also be translated and validated in multiple languages, as has been done for Pediatric Early Warning Systems (PEWS),^50^ promoting more widespread global use.

This study has several limitations. Although all beta‐testing sites completed the PROACTIVE assessment, they faced challenges including the time required, limited experience using online platforms, variable internet access, and difficulties assembling a local multidisciplinary team due to lack of staff fluency in English. Our team supported sites by delivering each PROACTIVE module through a stepwise, mentored approach over 4 to 6 weeks and provided some centers extra time (1–2 weeks) to complete the assessment. We also supported electronic data entry when needed and provided recorded videos and written instructions, allowing centers to move at their own pace. During feedback sessions, participants reported the length of the tool as reasonable and the mentorship for data collection and entry helpful for timely completion. To address language limitation and aid future centers recruit members from other disciplines who may not be fluent in English, we are planning to translate the PROACTIVE tool to multiple languages.

While the capacity and quality indicators included in the PROACTIVE tool were developed by a formal consensus‐process by POCC experts globally,^17^ their relationship to patient outcomes has not yet been empirically validated. Similarly, although a high‐median importance (≥7) in both relevance and actionability with ≥80% evaluator agreement was used as a criterion to select POCC indicators, the impact of these indicators on patient outcomes must be evaluated in future work. Additionally, we will be integrating data being collected by the St. Jude PrOFILE tool and SJCARES registry^51^ to analyze outcomes per cancer type and severity, and to validate components of PROACTIVE as they relate to patient outcomes. Importantly, 7 of the 11 beta‐testing centers reported collaborative work to compare outcomes for critically ill PHO patients between centers. This is not surprising as most of these centers actively collaborate with St. Jude Global in various initiatives to improve survival of pediatric cancer patients, including the multicenter PEWS program.^52^, ^53^

Finally, common POCC challenges identified in this pilot included 11‐centers and represent a cross‐sectional analysis at one time‐point. Pilot centers, however, manage high volumes of PHO patients with a median of 1200 admissions/year, represented a diversity of country income‐levels, and identified challenges are likely similar to other high‐volume centers. Currently, our team is scaling‐up PROACTIVE with a cohort of 19‐centers, with future plans for annual cohorts that pair data collection to structured quality improvement workshops to help hospitals identify and prioritize among multiple improvement opportunities. Hence, this work establishes PROACTIVE as a guided contextually appropriate institutional self‐assessment tool that can be used by centers once or every 1–2 years to monitor their progress over time. Among the 11 centers in this cohort, one opted to retake the assessment one year later to evaluate progress following implemented changes. We expect that this tool will be useful to all centers providing POCC care, including those in low‐, middle‐, and high‐income countries.

## 5 CONCLUSION

This work outlines the development and pilot testing of PROACTIVE, a pragmatic and feasible diagnostic tool to identify challenges in POCC services globally. Pilot testing of PROACTIVE demonstrated the usability, feasibility, and applicability of this tool in hospitals of varying resource levels and its aggregated data allowed for identification of common organizational challenges in POCC services. Global use of PROACTIVE in hospitals of all resource‐levels will help centers to effectively map local capacity and benchmark with similar centers at the local and regional level. Additionally, PROACTIVE allows for a thorough assessment of common global POCC challenges, promoting international collaborative research and development of cost‐effective quality improvement interventions to improve outcomes for children with cancer worldwide.

## AUTHOR CONTRIBUTIONS

**Anita V. Arias:** Conceptualization (lead); data curation (lead); formal analysis (lead); investigation (lead); methodology (lead); project administration (lead); resources (lead); software (lead); supervision (lead); validation (lead); visualization (lead); writing – original draft (lead); writing – review and editing (lead). **Firas M. Sakaan:** Data curation (equal); formal analysis (equal); investigation (equal); project administration (supporting); software (supporting); validation (equal); visualization (supporting); writing – review and editing (equal). **Maria F Puerto‐Torres:** Formal analysis (supporting); investigation (equal); project administration (supporting); software (equal); visualization (equal); writing – review and editing (equal). **Zebin Al Zebin:** Data curation (equal); formal analysis (supporting); investigation (equal); visualization (supporting); writing – review and editing (equal). **Parthasarathi Bhattacharyya:** Data curation (equal); formal analysis (supporting); investigation (equal); visualization (supporting); writing – review and editing (equal). **Adolfo Cardenas:** Data curation (equal); formal analysis (supporting); investigation (equal); visualization (supporting); writing – review and editing (equal). **Sanjeeva Gunasekera:** Data curation (equal); formal analysis (supporting); investigation (equal); visualization (supporting); writing – review and editing (equal). **Joyce B Kambugu:** Data curation (equal); formal analysis (supporting); investigation (equal); visualization (supporting); writing – review and editing (equal). **Kirill Kirgizov:** Data curation (equal); formal analysis (supporting); investigation (equal); visualization (supporting); writing – review and editing (equal). **Jaime Libes:** Data curation (equal); formal analysis (supporting); investigation (equal); visualization (supporting); writing – review and editing (equal). **Ruth Angelica Martinez Soria:** Data curation (equal); formal analysis (supporting); investigation (equal); visualization (supporting); writing – review and editing (equal). **Nune V. Matinyan:** Data curation (equal); formal analysis (supporting); investigation (equal); visualization (supporting); writing – review and editing (equal). **Alejandra Mendez Aceituno:** Data curation (equal); formal analysis (supporting); investigation (equal); visualization (supporting); writing – review and editing (equal). **Janet Middlekauff:** Formal analysis (supporting); project administration (supporting); software (equal); visualization (equal); writing – review and editing (equal). **Katie R. Nielsen:** Data curation (equal); formal analysis (supporting); investigation (equal); visualization (supporting); writing – review and editing (equal). **Andrew Pappas:** Formal analysis (supporting); project administration (supporting); software (equal); visualization (equal); writing – review and editing (equal). **Hong Ren:** Data curation (equal); formal analysis (supporting); investigation (equal); visualization (supporting); writing – review and editing (equal). **Rana Sharara‐Chami:** Data curation (equal); formal analysis (supporting); investigation (equal); visualization (supporting); writing – review and editing (equal). **Silvio F. Torres:** Data curation (equal); formal analysis (supporting); investigation (equal); visualization (supporting); writing – review and editing (equal). **Jennifer McArthur:** Data curation (equal); formal analysis (supporting); investigation (equal); visualization (supporting); writing – review and editing (equal). **Asya Agulnik:** Conceptualization (equal); formal analysis (equal); funding acquisition (lead); investigation (equal); methodology (equal); project administration (supporting); supervision (lead); validation (equal); visualization (equal); writing – original draft (equal); writing – review and editing (equal).

## FUNDING INFORMATION

This study was supported by the American Lebanese Syrian Associated Charities (ALSAC).

## CONFLICT OF INTEREST

The authors have no conflict of interest to disclose.

## PRECIS FOR USE IN THE TABLE OF CONTENTS

PROACTIVE is a contextually appropriate diagnostic tool to assess capacity and quality of care in pediatric onco‐critical care services among hospitals with variable resources, and can help clinicians prioritize and develop tailored interventions to strengthen pediatric onco‐critical care services and outcomes globally.

## Supporting information


Appendix S1

## Data Availability

The data that support the findings of this study are available from the corresponding author upon reasonable request.
