# Supplementary material for: Development and pilot testing of PROACTIVE: A pediatric onco‐critical care capacity and quality assessment tool for resource‐limited settings
Source: Cancer Med. 2022 Nov 2;12(5):6270–82. doi: 10.1002/cam4.5395 (PMC10028058; doi:10.1002/cam4.5395)
Supplement: Supplementary file 1 — Appendix S1 [file CAM4-12-6270-s001.docx]

**SUPPLEMENTAL MATERIAL**

**DEVELOPMENT AND PILOT TESTING OF PROACTIVE: A PEDIATRIC ONCO-CRITICAL CARE CAPACITY AND QUALITY ASSESSMENT TOOL FOR RESOURCE-LIMITED SETTINGS**

**Supplementary Appendix**

| **Section** | **Page** |
| --- | --- |
| **eTable 1. Framework of Domains and Subdomains** | **2** |
| **eTable 2. Type of questions with assigned scores per answer** | **3** |
| **eFigure 1. Participating hospitals by country and World Bank income category** | **4** |
| **eFigure 2. PROACTIVE phases** | **5** |
| **eTable 3. Content and areas evaluated by beta-testing site leaders** | **6** |
| **eTable 4. Oncology questions included from the PrOFILE tool** | **7** |
| **eTable 5. Number of indicators/questions per stage of development of PROACTIVE** | **8** |
| **eTable 6. Demographics and characteristics of beta-testing site respondents** | **9** |
| **eFigure 3. PROACTIVE report (sample)** | **10** |
| **eTable 7 – Table of removed and added questions per Beta-testing site recommendations** | **11-12** |
| **eTable 8. PROACTIVE Tool** | **13-37** |
| **Supplemental References** | **38** |

**eTable 1. Framework of Domains and Subdomains**

| **Domains** | **Sub-domains** |
| --- | --- |
| 1. **National Context** | 1. National Profile & Training Programs |
| 1. **Facility and Local Context** | 1. Configuration of Services and Facility Utilities |
| 1. **Personnel** | 1. Core Team |
|  | 1. Core Consultants |
|  | 1. Staffing |
|  | 1. Education, Research and Quality Improvement |
| 1. **Service Capacity** | 1. Service Organization |
|  | 1. Acute Management of Critically ill PHO patients |
|  | 1. Pediatric Outreach Team |
|  | 1. Standard Precautions and Infection Prevention |
|  | 1. Guidelines and Protocols |
| 1. **Service Integration** | 1. Communication and Multidisciplinary Care |
|  | 1. Parent Involvement |
|  | 1. Quality Indicators |
| 1. **Supportive Services** | 1. General Supportive Services |
|  | 1. General Laboratory |
|  | 1. Imaging Services |
|  | 1. Blood Bank/Transfusion Services |
|  | 1. OR/Anesthesia |
| 1. **Medication and Equipment** | 1. Medications |
|  | 1. Equipment and Supplies |
| 1. **Outcomes** | 1. Diagnosis &Outcomes |

**eTable 2. Type of questions with assigned scores per answer**

| **Type of Questions** | **Assigned Scores per Answer** |
| --- | --- |
| Dichotomus | \| **Answers** \| **Score** \| \| --- \| --- \| \| Yes \| 5 \| \| No \| 0 \| \| Do not know/No data available \| 0 \| |
| Likert Scale | 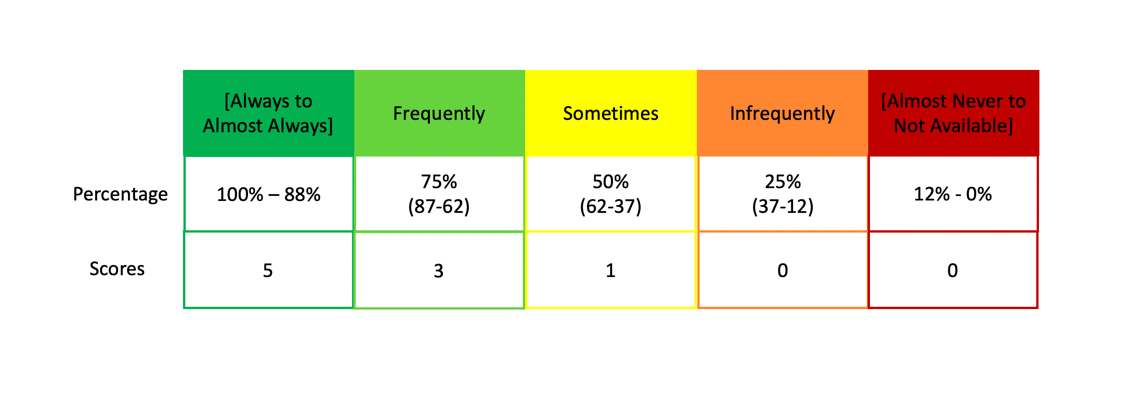 |

**eFigure 1. Participating hospitals by country and World Bank income category**^1^


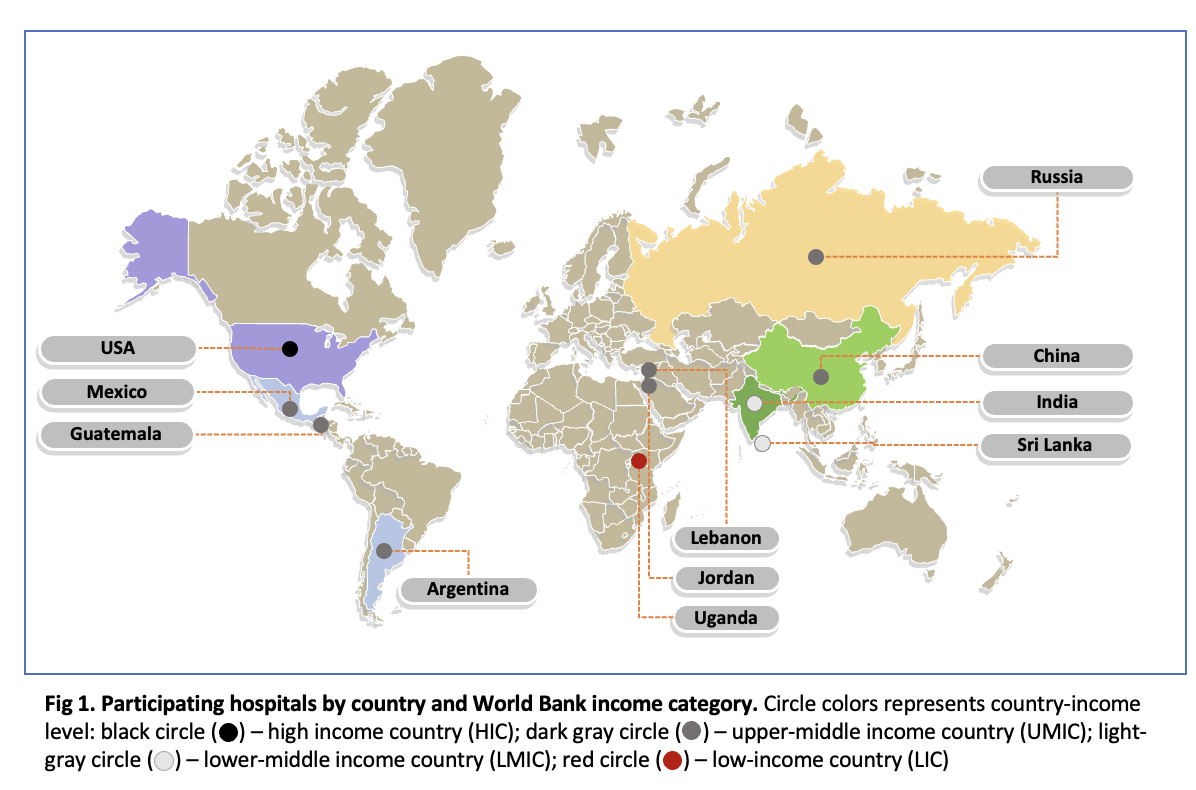


Circle colors represents country-income level:

- Black circle ( ) – high income country (HIC)
- Dark gray circle ( ) – upper-middle income country (UMIC)
- Light-gray circle ( ) – lower-middle income country (LMIC)
- Red circle ( ) – low-income country (LIC).

**eFigure 2. PROACTIVE phases**

**
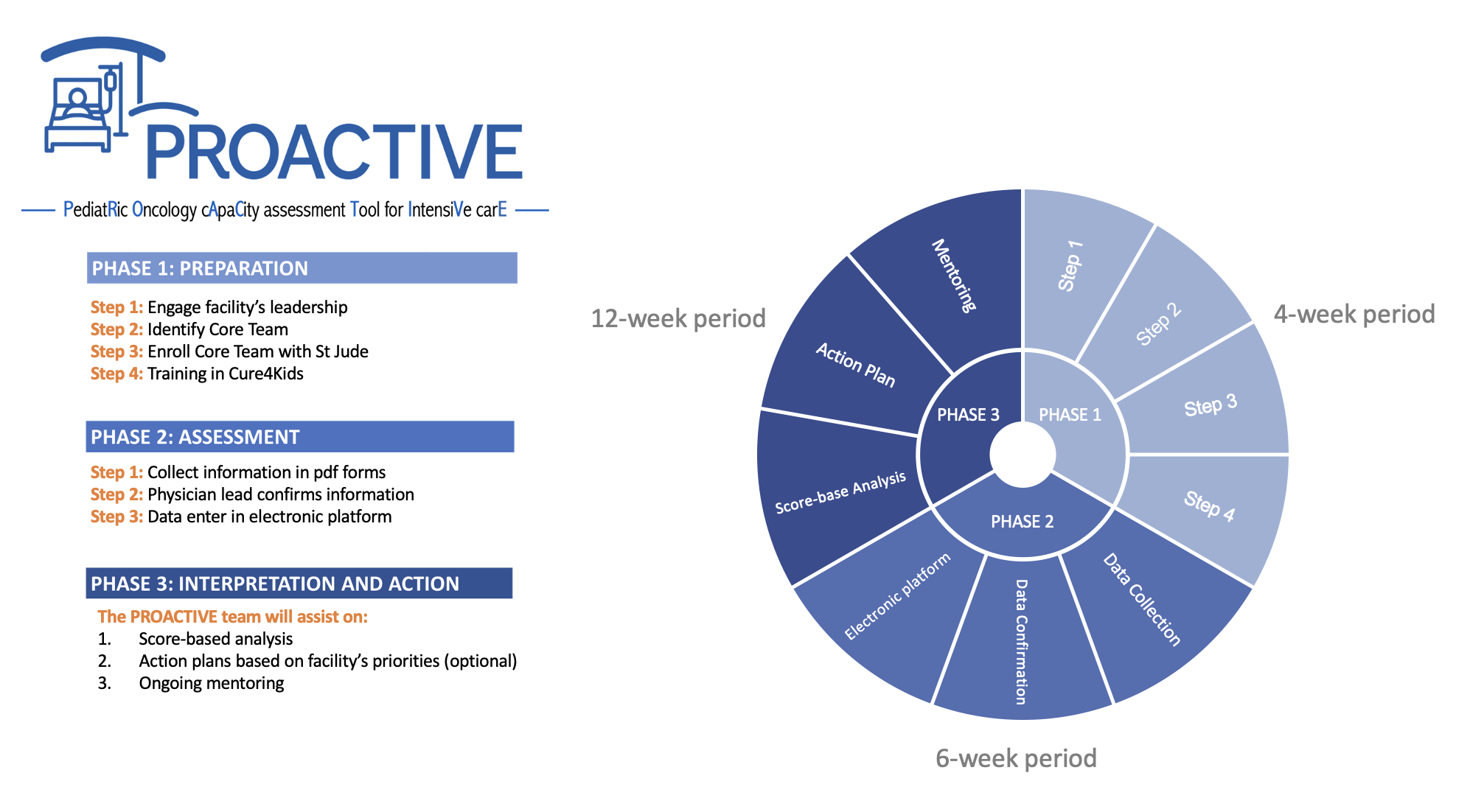
**

*Format of the PROACTIVE phases are based on the PrOFILE tool^2^

**eTable 3. Content and areas evaluated by beta-testing site leaders**

|  | **Main area evaluated** | **Content evaluated for challenges and suggestions for improvement** |
| --- | --- | --- |
| **Survey 1** | Introductory meetings | Presentations: content, relevance, clarity, organization, structure, and length |
|  | Internal Hospital Process | Engagement of hospital Leadership, gathering of Core Team and IRB process |
|  | User Manual | User’s manual: content, organization, and structure |
|  | Educational Sessions for the Workflow | Presentations for each phase (preparation, assessment, and interpretation and action): content, relevance, clarity, organization, structure, and length |
| **Survey 2** | PROACTIVE Tool | Registration information, tool’s organization and structure in REDCap and its usability. All questions were also included in the survey and each one was evaluated for content, clarity and relevance, challenges and suggestions for improvement were requested. |

**eTable 4 – Oncology questions included from the PrOFILE tool**^2^

| Facility and Local Context | 1. Which of the following best describes your facility? 1 = Pediatric Hematology and/or Oncology Hospital, 2 = Cancer Hospital or Institute, 3 = Children's Hospital, 4 = General Hospital, 5 = Other 2. Is your facility public or private? 3. Is your facility a government-designated teaching or training facility? 4. What is the maximum age (in years) for pediatric services at your facility? 5. What is the total # of inpatient beds in your facility? |
| --- | --- |
| Configuration of Services | 1. Total # of general pediatric inpatient beds in your facility? 2. Is the PHO inpatient care area a separate unit or ward? 3. What is the total # of inpatient pediatric hematology-oncology (PHO) beds? 4. What is the average (Range) Number of PHO patients in each hospital room? 5. What is the total # of single-person infection isolation rooms available for PHO patients? 6. Does your facility perform pediatric bone marrow/stem cell transplants? 7. What is the total # of bone marrow/stem cell transplant beds available for PHO patients? |
| Service Capacity | 1. What is the average # of total pediatric hospital admissions per year? 2. What is the average # of PHO admissions per year to your facility? 3. Average # of bone marrow transplant patient admitted per year to your facility? |
| Patient and Outcomes | 1. What is the total # of newly diagnosed pediatric cancer patients treated at your facility in the past year? 2. What is the total # of bone marrow/stem cell transplants performed at your facility in the past year? |

**eTable 5. Number of Indicators/Questions per stage of development of PROACTIVE**

| **Domain** | **Subdomains** | **# Indicators** | **Alpha-Testing**  **(# questions)** | **Beta-testing**  **(# questions/questions per survey)** | **Final Tool** |
| --- | --- | --- | --- | --- | --- |
| National Context | National Profile and Training Programs | 3 | 3 | 3 (3 PICU) | 4 PICU |
| Facility & Local Context | Configuration of services and Facility utilities | 7 | 22 | 25 (6 PICU, 14 ONC, 5 Both) | 21 (7 PICU, 14 ONC) |
| Personnel | Core Team | 4 | 3 | 3 (1 PICU, 2 Both) | 3 PICU |
|  | Core Consultants | 3 | 3 | 3 (3 Both) | 4 PICU |
|  | Staffing | 5 | 4 | 4 (3 PICU, 1 Both) | 6 PICU |
|  | Education, Research and QI | 6 | 7 | 7 (6 PICU, 1 Both) | 7 PICU |
| Service Capacity | Service Organization | 3 | 3 | 3  (2 PICU, 1 Both) | 3 PICU |
|  | Acute management of Critically ill PHO patients | 3 | 5 | 5 (5 Both) | 6 (3 PICU, 3 ONC) |
|  | Pediatric Outreach Team | 6 | 6 | 6 (6 Both) | 6 ONC |
|  | Standard Precautions | 4 | 10 | 10 (10 PICU) | 11 PICU |
|  | Guidelines and Protocols | 13 | 13 | 13 (8 PICU, 5 Both) | 14 (9 PICU, 5 ONC) |
| Service Integration | Communication and Multidisciplinary care | 7 | 8 | 8 (6 PICU, 2 Both) | 9 (5 PICU, 4 ONC) |
|  | Parent involvement | 2 | 2 | 3 (1 PICU, 2 Both) | 5 (3 PICU, 2 ONC) |
|  | Quality Indicators | 5 | 4 | 5 (4 PICU, 1 Both) | 12 PICU |
| Supportive Services | General Supportive Services | 7 | 7 | 7 (6 PICU, 1 Both) | 7 PICU |
|  | General Labs | 6 | 13 | 14 (14 PICU) | 13 PICU |
|  | Imaging Services | 7 | 7 | 7 (7 PICU) | 7 (4 PICU, 3 ONC) |
|  | Blood Bank | 3 | 9 | 8 (8 PICU) | 8 PICU |
|  | OR/Anesthesia | 2 | 2 | 2 (2 PICU) | 2 PICU |
| Meds & Equipment | Medications | 11 | 11 | 11(11PICU) | 11 (6 PICU, 5 ONC) |
|  | Equipment and Supplies | 8 | 26 | 26 (26 PICU) | 26 (23 PICU, 3 ONC) |
| Outcomes | Diagnosis and Outcomes | 4 | 8 | 9 (3 PICU, 4 ONC, 2 Both) | 15 (8 PICU, 7 ONC) |
| **Total** | | **119 + 17 ONC** | **176** | **182 (164 PICU, 55 ONC)** | **200 (148 PICU, 52 ONC)** |

**eTable 6 – Demographics and characteristics of Beta-testing site respondents (n=22)**

| **Category** | | **Beta-testing (n, %)** |
| --- | --- | --- |
| **Total** | Participants | 22 (100%) |
| Gender | Female  Males | 16 (73%)  6 (27%) |
| Specialty | Pediatric Intensivists  Anesthesiologist/Intensivists  Pediatric Oncologists  Pediatricians  Nurses | 7 (32%)  2 (9%)  8 (36%)  3 (14%)  2 (9%) |
| Years of Experience | ≤ 5 years  6-10 years  11-15  16-19  ≥ 20 | 6 (27%)  8 (36%)  3 (14%)  1 (5%)  4 (18%) |
| Region | North America  Latin America and the Caribbean  Europe and Central Asia  East Asia and Pacific  South Asia  Middle East and North Africa  Sub-Saharan Africa | 2 (9%)  6 (27%)  2 (9%)  2 (9%)  4 (18%)  4 (18%)  2 (9%) |

**eFigure 3. PROACTIVE Report (sample)**

**
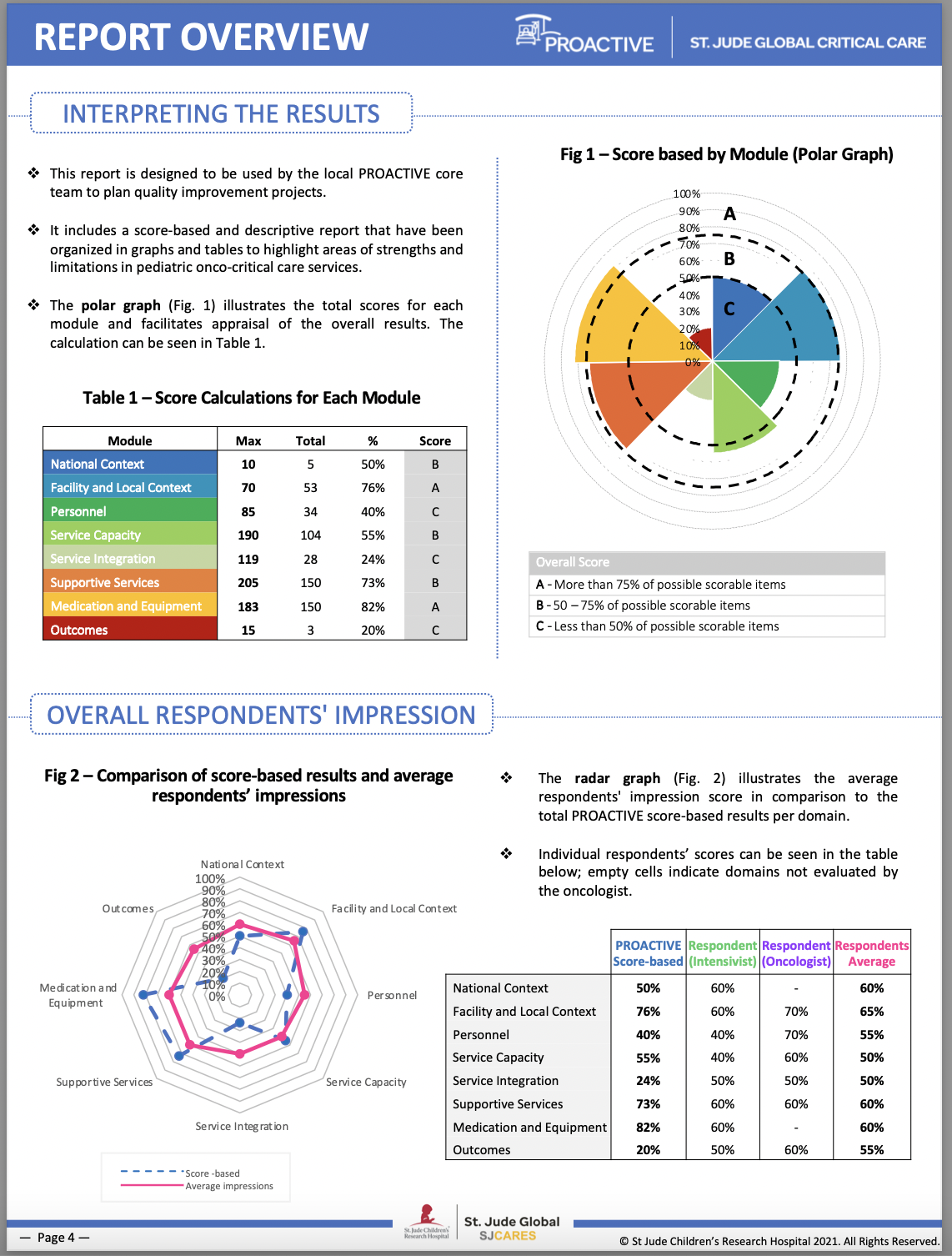

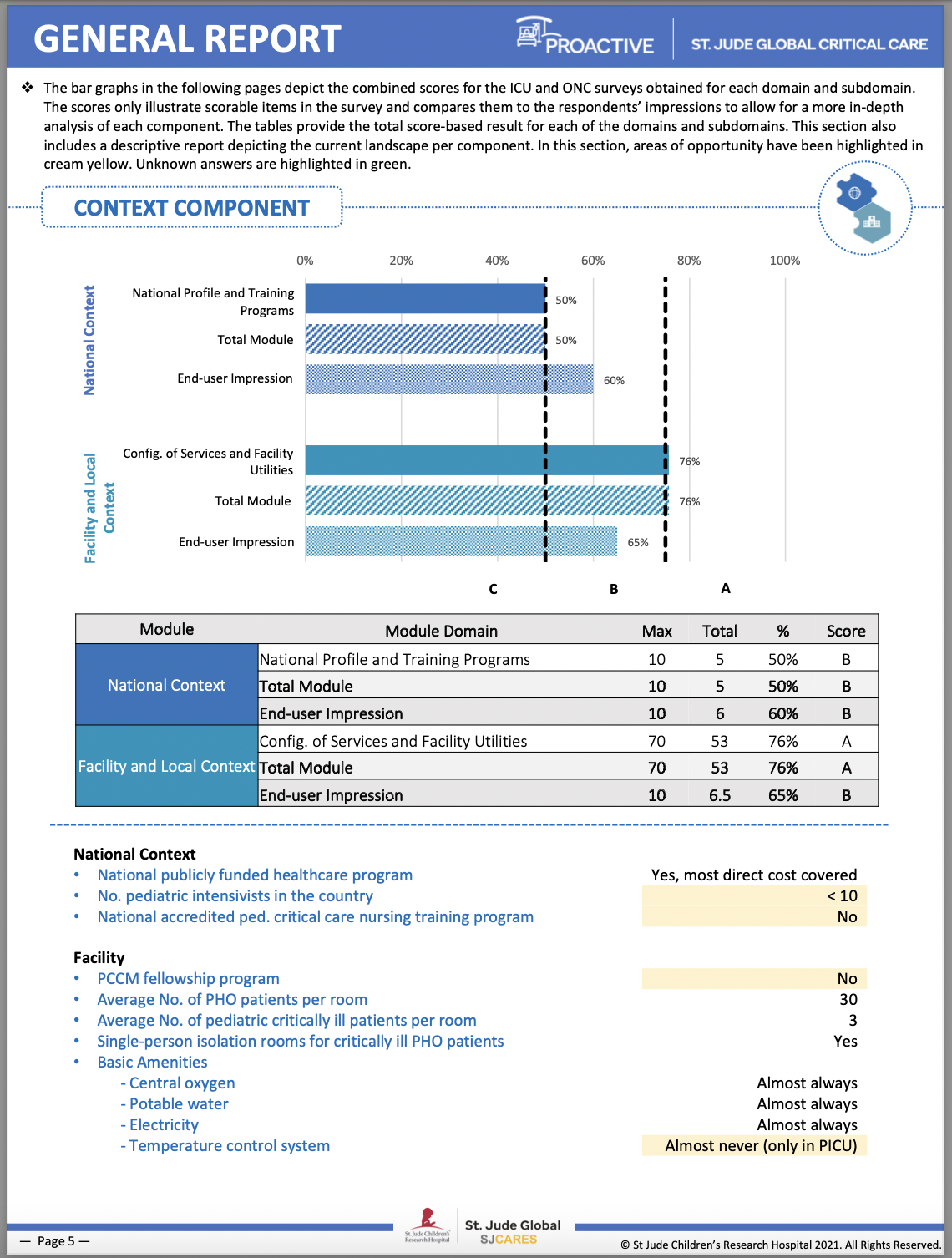

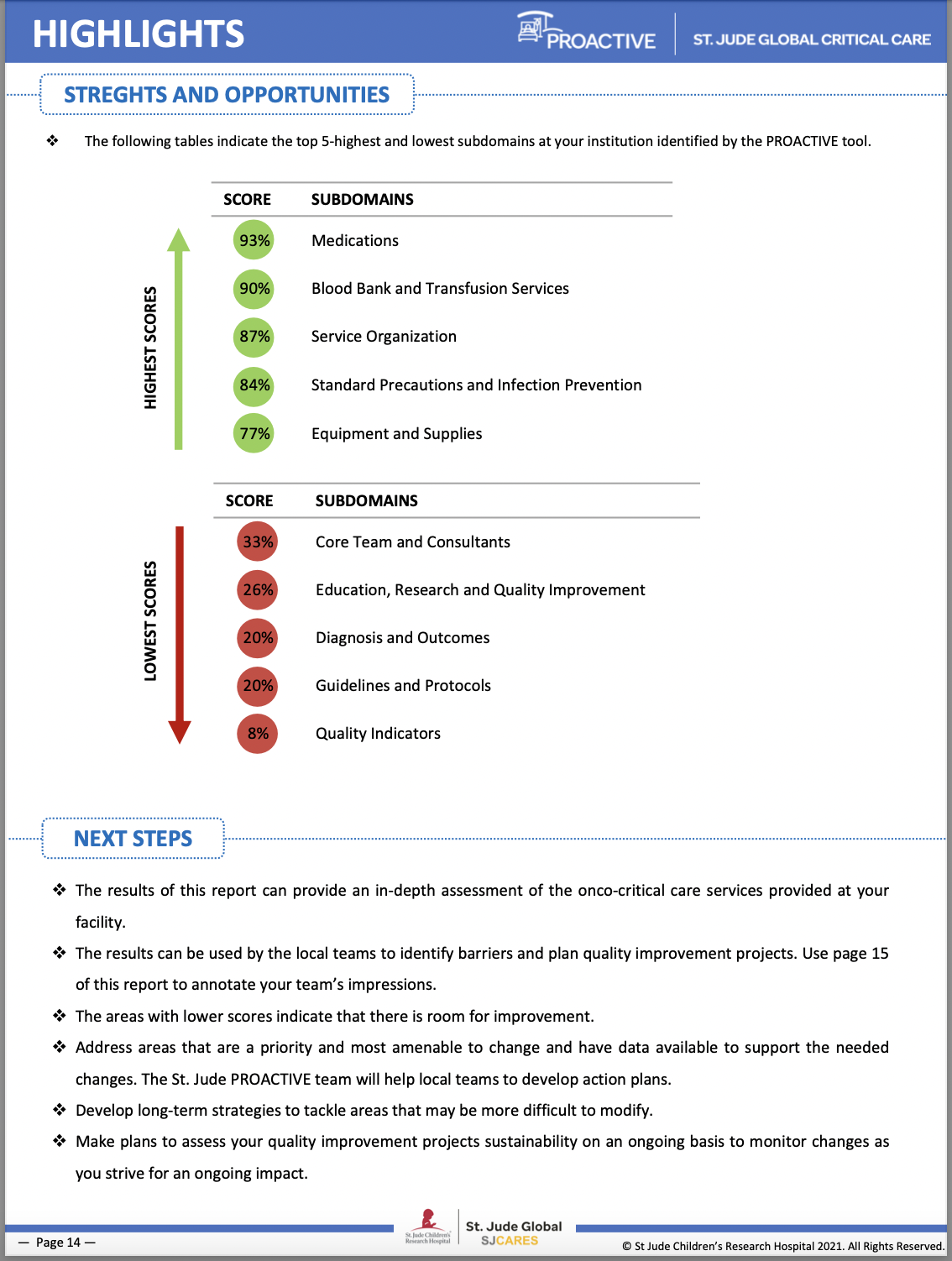
**

**eTable 7 – Table of removed and added questions per Beta-testing site recommendations**

| **Removed Questions per Domain and Survey** | | |
| --- | --- | --- |
| **Domain** | **Survey** | **Question** |
| **Facility and Local Context** | PICU | 1. What is the average number of pediatric critical care patients in each room in the PICU (or area where critically ill patients are treated)? |
|  | ONC | 1. What is the average number of PHO patients in each hospital room? |
|  | ONC | 1. What is the total number of single-person infection isolation rooms available for PHO patients at your facility? (NOT including PICU beds) |
| **Added Questions per Domain and Survey** | | |
| **Domain** | **Survey** | **Question** |
| **National Context** | PICU | 1. Does your country have an accredited pediatric critical care training or certification program for physicians (fellowship in PCCM)? |
| **Facility and Local Context** | ONC | 1. Does your hospital have an accredited pediatric hematology-oncology) fellowship program? |
| **Personnel - Core Consultants** | PICU | 1. How often are pediatric critical care physician available for in-person consults on critically ill PHO patients? (Some consults can be done by phone, but consultants examine patients within 24 hrs. after being consulted) |
| **Personnel -Staffing** | PICU | 1. What is the nurse-to-patient ratio in the PICU (or area where critically ill PHO patients are treated) during day-time shifts, 7 days a week? (DURING the COVID-19 pandemic) |
|  | PICU | 1. What is the nurse-to-patient ratio in the PICU (or area where critically ill PHO patients are treated) during night-time shifts, 7 days a week? (DURING the COVID-19 pandemic) |
| **Service Capacity - Acute mgmt. of CIPHOP patients** | PICU | 1. Is there a system (operational plan) in place to transfer critically ill PHO patients to other hospitals if NO beds are expected to become available in the PICU (or areas where critically ill PHO patients are treated) at your hospital? |
| **Service Capacity - Standard Precautions** | PICU | 1. Is there a standardized process to monitor adherence to hand hygiene protocols by healthcare personnel at your hospital? |
| **Service Capacity - Guidelines and Protocols** | PICU | 1. Is there a clinical guideline (updated with the latest medical evidence) for the management and treatment of blood products transfusion reactions or transfusion related complications in critically ill PHO patients? |
| **Service Integration – Comm. and Multidisc. Care** | ONC | 1. How often are palliative care services available when needed for the care of critically ill PHO patients? |
|  | ONC | 1. How often are psychological/emotional support services available when needed for families of critically ill PHO patients? |
| **Service Integration - Parent Involvement** | PICU | 1. Does your hospital have a system to measure parental satisfaction with the quality of care provided to their child in the PICU or area where critically ill PHO patients are treated? |
|  | ONC | 1. How often are restrictions in the number of visitors and timing for visits removed for critically ill patients at the end-of life? (PRIOR to COVID) |
| **Service Integration - Quality Indicators** | PICU | 1. Does your hospital have a system in place to evaluate the daily need to continue or remove devices (central lines, urine catheters, etc.) in critically ill patients (including critically ill PHO patients)? |
|  |  | 1. Does you hospital have a protocol to prevent deep venous thrombosis in critically ill patients (including critically ill PHO patients)? |
|  |  | 1. Does you hospital have a protocol to prevent peptic ulcer in critically ill patients (including critically ill PHO patients)? |
|  |  | 1. Does you hospital have a protocol to perform daily oral care in critically ill patients (including critically ill PHO patients)? |
|  |  | 1. Does your hospital perform spontaneous breathing trials in mechanically ventilated patients (including critically ill PHO patients)? |
|  |  | 1. Does your hospital have a system in place to evaluate the daily need to continue or discontinue current antimicrobial treatment in critically ill patients (including critically ill PHO patients)? |
|  |  | 1. Does your hospital have a system in place to evaluate the daily need to continue, discontinue or change current sedation medications in critically ill pediatric patients (including critically ill PHO patients)? |
| **Outcomes** | ONC | 1. How often are multidisciplinary meetings to review unexpected transfers, clinical incidents, or near misses for critically ill PHO patients conducted at your facility? |
|  | PICU | 1. How often are multidisciplinary meetings conducted to review resource utilization (e.g., mechanical ventilation days, urinary catheter days, iNO days, etc.) at your hospital? PICU |
|  | PICU | 1. What is the total number of pediatric patient admitted to the PICU/IMCU/HDU (or area where critically ill patients are managed) during the past 12 months? (At end of this module provide in the comments the data source) PICU |
|  | PICU | 1. What is the total number of pediatric patient who have died in the PICU/IMCU/HDU (or area where critically ill patients are managed) during the past 12 months? (At end of this module provide in the comments the data source) PICU |
|  | PICU | 1. What is the total number of PHO/BMT patients who have died of sepsis in the PICU/IMCU/HDU (or area where critically ill patients are managed) during the past 12 months? (At end of this module provide in the comments the data source) PICU |
|  | PICU | 1. What is the total number of PHO/BMT patients who have died of respiratory failure in the PICU/IMCU/HDU (or areas where critically ill patients are managed) during the past 12 months? (At end of this module provide in the comments the data source) |

**Abbreviations**: BMT (Bone Marrow Transplant); CIPHOP (Critically ill Pediatric Hematology-Oncology Patients); HDU (High-Dependency Unit); IMCU (Intermediate Medical Care Unit); iNO (inhaled Nitric Oxide); ONC (Oncology); PCCM (Pediatric Critical Care Medicine); PHO (Pediatric Hematology-Oncology); PICU (Pediatric Intensive Care Unit).

**eTable 8. PROACTIVE Tool.** Final questions included in the PROACTIVE tool. This table shows the main questions, answer choices and their designation to PICU or Oncology surveys.

| **Domain** | **Sub-domain** | **Survey** | **Questions** | **Answer Choices** | **Scores** |
| --- | --- | --- | --- | --- | --- |
| National Context | National Profile & Training Programs | PICU | 1. How many formally trained Pediatric Critical Care Physicians are currently practicing in your country? (In the comments provide data source; this information might be available at your country’s critical care or pediatric societies registries) | a) 1-499 | N/A |
|  |  |  |  | b) 500-999 |  |
|  |  |  |  | c) 1000-1499 |  |
|  |  |  |  | d) 1500-1999 |  |
|  |  |  |  | e) 2000-2499 |  |
|  |  |  |  | f) 2500-2999 |  |
|  |  |  |  | g) ≥ 3000 |  |
|  |  |  |  | h) Do not know/No data available |  |
|  |  |  | 2. Does your country have an accredited pediatric critical care training or certification program for physicians (fellowship in pediatric critical care medicine)? | a) Yes | 5 |
|  |  |  |  | b) No, where do interested physicians typically obtain their training? | 0 |
|  |  |  | 3. Does your country have an accredited pediatric critical care nursing training or certification program? | a) Yes | 5 |
|  |  |  |  | b) No, where do interested nurses typically obtain their training? | 0 |
|  |  |  | 4. Does your country have a national publicly funded healthcare program (endorsed by the Ministry of Health) that provides coverage for pediatric critical illness? (Please indicate your country’s funding sources) | a) Most direct costs of care are covered; residual indirect costs exist and can cause material hardship, but pose low additional risk of family bankruptcy | 5 |
|  |  |  |  | b) Funding mechanism for most direct costs exists, but families remain highly dependent on alternate sources for coverage of indirect costs | 3 |
|  |  |  |  | c) No funding mechanism exists, but non-public sources are available; families are highly dependent on these alternate sources of funding for coverage of direct and indirect costs | 1 |
|  |  |  |  | d) No funding mechanism exists AND no significant input from non-public sources; families heavily burden by out-of-pocket expenses | 0 |
| Facility and Local Context | Configuration of services and Facility Utilities | PICU | 1. Is your hospital a government-designated teaching or an accredited training facility? | a) Yes | 5 |
|  |  |  |  | b) No | 0 |
|  |  |  | 2. Does your hospital have an accredited pediatric critical care medicine (PCCM) fellowship program? | a) Yes | 5 |
|  |  |  |  | b) No | 0 |
|  |  |  | 3. Is there a designated Pediatric Intensive Care Unit (PICU), separated from other inpatient locations (e.g., general ward) in your hospital? | a) Yes | 5 |
|  |  |  |  | b) No | 0 |
|  |  |  | 3a. If the answer is NO for question 3, which of the following best describes the area where critically ill pediatric patients are treated? | a) Pediatric High Dependency Unit (HDU) or Pediatric Intermediate Care Unit (IMCU) | 4 |
|  |  |  |  | b) Adult ICU (mixed unit of pediatric and adult patients) | 3 |
|  |  |  |  | c) General pediatric ward | 2 |
|  |  |  |  | d) Combined pediatric/adult ward | 1 |
|  |  |  |  | e) Other, please describe: | 0 |
|  |  |  | 4. What is the total number of pediatric intensive care (PICU) beds available at your hospital? (NOT including IMCU/HDU or NICU beds) | a)  Prior to COVID: ________ (number) | N/A |
|  |  |  |  | b)  Now: ________ (number) |  |
|  |  |  | 5. What is the total number of intermediate care (IMCU) pediatric or high dependency unit (HDU) beds available in your hospital? | a)  Prior to COVID: ________ (number) | N/A |
|  |  |  |  | b)  Now: ________ (number) |  |
|  |  |  | 6. Are single-person infection isolation rooms available for critically ill PHO patient admitted to the PICU (or area where critically ill patients are treated)? | a)  Yes | 5 |
|  |  |  |  | b)  No | 0 |
|  |  |  | 7. Does your hospital have centrally supplied oxygen and/or compressed air administration systems available? | a) Always (24 hrs. a day/7 day a week or 100% of the time) | 5 |
|  |  |  |  | b) Almost Always (80-99% of the time) | 4 |
|  |  |  |  | c) Frequently (60-79% of the time) | 3 |
|  |  |  |  | d) Sometimes/Moderate availability (41-59% of the time) | 2 |
|  |  |  |  | e) Infrequently/Limited availability (21-40% of the time) | 1 |
|  |  |  |  | f)  Almost Never (1-20% of the time) | 0 |
|  |  |  |  | g) Never/Not available | 0 |

| Facility and Local Context | Configuration of services and Facility Utilities | PICU | 7a. If NOT Always/Almost Always for question 7, are the breaks in oxygen supply ≤ 2hrs per day? | a)  Yes | 1 |  |
| --- | --- | --- | --- | --- | --- | --- |
|  |  |  |  | b)  No | 0 |  |
|  |  | ONC | 1. Which of the following best describes your hospital? | a)  Children’s hospital | N/A |  |
|  |  |  |  | b)  Pediatric Hematology and/or Oncology Hospital |  |  |
|  |  |  |  | c)  Cancer Hospital or Institute (adult and pediatric) |  |  |
|  |  |  |  | d)  General Hospital (adult and pediatric) |  |  |
|  |  |  |  | e)  Women’s and Children’s Hospital |  |  |
|  |  |  |  | f)  Other, please describe: |  |  |
|  |  |  | 2. Is your hospital public or private? | a)  Public | 5 |  |
|  |  |  |  | b)  Private, not-for-profit | 5 |  |
|  |  |  |  | c)  Private, for-profit | 3 |  |
|  |  |  |  | d)  Other, please describe: | 3 |  |
|  |  |  | 3. Does your hospital have an accredited pediatric hematology-oncology) fellowship program? | a)  Yes | 5 |  |
|  |  |  |  | b)  No | 0 |  |
|  |  |  | 4. What is the maximum patient age (in years) for pediatric services at your hospital? | a)  ________ (years) | N/A |  |
|  |  |  | 5. What is the total number of inpatient beds (adult and/or pediatric) at your hospital? (Including critical care and neonatal intensive care [NICU] beds) | a)  Prior to COVID: ________ (number) | N/A |  |
|  |  |  |  | b)  Now: ________ (number) |  |  |
|  |  |  | 6. What is the total number of pediatric inpatient beds at your hospital? (Including pediatric critical care beds but excluding NICU beds) | a)  Prior to COVID: ________ (number) | N/A |  |
|  |  |  |  | b) Now: ________ (number) |  |  |
|  |  |  | 7. Is the PHO inpatient care area a separate unit or ward? | a)  Yes | 5 |  |
|  |  |  |  | b) No | 0 |  |
|  |  |  | 7a. If the answer is NO for question 7, which of the following best describe the setting: | a) General pediatrics ward | 3 |  |
|  |  |  |  | b) Oncology ward (adult and pediatric combined) | 2 |  |
|  |  |  |  | c) General ward (adult and pediatric combined) | 1 |  |
|  |  |  |  | d) Other, please describe: | 0 |  |
|  |  |  | 8. What is the total number of inpatient pediatric hematology-oncology (PHO) beds? (NOT including PICU and NICU beds) | a) Prior to COVID: ________ (number) | N/A |  |
|  |  |  |  | b) Now: ________ (number) |  |  |
|  |  |  | 9. Does your hospital perform pediatric bone marrow/stem cell transplants? (Check all that apply) | a) Autologous transplant | 5 |  |
|  |  |  |  | b) Allogenic transplant | 5 |  |
|  |  |  |  | c) Not available | 0 |  |
|  |  |  | 9a. Is the pediatric bone marrow/stem cell transplant (BMT) area a separate unit or ward? | a) Yes | 5 |  |
|  |  |  |  | b) No | 0 |  |
|  |  |  | 9b. If the answer is NO for question 9a, which of the following best describes the setting: | a) PHO ward | 4 |  |
|  |  |  |  | b) General pediatrics ward | 3 |  |
|  |  |  |  | c) Oncology ward (adult and pediatric combined) | 2 |  |
|  |  |  |  | d) General ward (adult and pediatric combined) | 1 |  |
|  |  |  |  | e) Other, please describe: | 0 |  |
|  |  |  | 9c. What is the total number of bone marrow/stem cell transplant beds available for PHO patients at your hospital? | a) Prior to COVID: ________ (number) | N/A |  |
|  |  |  |  | b) Now: ________ (number) |  |  |
|  |  |  | 10. Does your hospital have electricity available? | a) Always (24 hrs. a day/7 day a week or 100% of the time) | 5 |  |
|  |  |  |  | b) Almost Always (80-99% of the time) | 4 |  |
|  |  |  |  | c) Frequently (60-79% of the time) | 3 |  |
|  |  |  |  | d) Sometimes/Moderate availability (41-59% of the time) | 2 |  |
|  |  |  |  | e) Infrequently/Limited availability (21-40% of the time) | 1 |  |
|  |  |  |  | f) Almost Never (1-20% of the time) | 0 |  |
|  |  |  |  | g) Never/Not available | 0 |  |
|  |  |  | 10a. If NOT Always/Almost Always for question 10, are the breaks in electricity ≤ 2hrs per day? | a)  Yes | 1 |  |
|  |  |  |  | b) No | 0 |  |

| Facility and Local Context | Configuration of services and Facility Utilities | ONC | 11. Does your hospital have functional back-up generators available to provide electricity in the event of power interruption? | a) Yes | 5 |  |
| --- | --- | --- | --- | --- | --- | --- |
|  |  |  |  | b) No | 0 |  |
|  |  |  |  | c) Do not know | 0 |  |
|  |  |  | 12. Does your hospital have potable water (drinking water supply or bottled/bulked water) available? | a) Always (24 hrs. a day/7 day a week or 100% of the time) | 5 |  |
|  |  |  |  | b) Almost Always (80-99% of the time) | 4 |  |
|  |  |  |  | c) Frequently (60-79% of the time) | 3 |  |
|  |  |  |  | d) Sometimes/Moderate availability (41-59% of the time) | 2 |  |
|  |  |  |  | e) Infrequently/Limited availability (21-40% of the time) | 1 |  |
|  |  |  |  | f) Almost Never (1-20% of the time) | 0 |  |
|  |  |  |  | g) Never/Not available | 0 |  |
|  |  |  | 12a. If NOT Always/Almost always for question 12, are the breaks in water supply ≤ 2hrs per day? | a) Yes | 1 |  |
|  |  |  |  | b) No | 0 |  |
|  |  |  | 13. Does your hospital have an emergency water supply alternative available to sustain hospital operations in case of service interruptions? (e.g., damage to main supply line) | a) Yes | 5 |  |
|  |  |  |  | b) No | 0 |  |
|  |  |  |  | c) Do not know | 0 |  |
|  |  |  | 14. Does your hospital have a functional environmental temperature control system (e.g., heater and/or air conditioner) available? (In the comments explain if it is ONLY available in certain areas of your hospital) | a) Always (24 hrs. a day/7 day a week or 100% of the time) | 5 |  |
|  |  |  |  | b) Almost Always (80-99% of the time) | 4 |  |
|  |  |  |  | c) Frequently (60-79% of the time) | 3 |  |
|  |  |  |  | d) Sometimes/Moderate availability (41-59% of the time) | 2 |  |
|  |  |  |  | e) Infrequently/Limited availability (21-40% of the time) | 1 |  |
|  |  |  |  | f) Almost Never (1-20% of the time) | 0 |  |
|  |  |  |  | g) Never/Not available | 0 |  |
|  |  |  | 14a. If NOT Always/Almost always for question 14, are the breaks in temperature control system ≤ 2hrs per day? | a) Yes | 1 |  |
|  |  |  |  | b) No | 0 |  |
| Personnel | Core Team | PICU | 1. Is a pediatric critical care physician part of the primary medical team responsible for the care of critically ill PHO patients? (Provides daily care and is not a consultant) | a) Always (100% of the time or 24 hrs. a day/7 day a week) | 5 |  |
|  |  |  |  | b) Almost Always (80-99% of the time or 24 hrs. Monday-Friday and some hours on the weekends) | 4 |  |
|  |  |  |  | c) Frequently (60-79% of the time or 24 hrs. Monday-Friday) | 3 |  |
|  |  |  |  | d) Sometimes/Moderate availability (41-59% of the time or only daytime 5-7 days a week) | 2 |  |
|  |  |  |  | e) Infrequently/Limited availability (21-40% of the time or only daytime 3-4 days a week) | 1 |  |
|  |  |  |  | f) Almost Never (1-20% of the time or only daytime 1-2 days a week) | 0 |  |
|  |  |  |  | g) Never/Not available | 0 |  |
|  |  |  | 1a. What is the total number of pediatric critical care physicians working at your hospital? | a) ________ (number) | N/A |  |
|  |  |  |  |  |  |  |
|  |  |  | If the answer is G for question 1 (NO pediatric intensivist available):  1b. Is a pediatrician with experience managing critically ill PHO patients part of the primary medical team? | a) Always (100% of the time or 24 hrs. a day/7 day a week) | 5 |  |
|  |  |  |  | b) Almost Always (80-99% of the time or 24 hrs. Monday-Friday and some hours on the weekends) | 4 |  |
|  |  |  |  | c) Frequently (60-79% of the time or 24 hrs. Monday-Friday) | 3 |  |
|  |  |  |  | d) Sometimes/Moderate availability (41-59% of the time or only daytime 5-7 days a week) | 2 |  |
|  |  |  |  | e) Infrequently/Limited availability (21-40% of the time or only daytime 3-4 days a week) | 1 |  |
|  |  |  |  | f) Almost Never (1-20% of the time or only daytime 1-2 days a week) | 0 |  |
|  |  |  |  | g) Never/Not available | 0 |  |
|  |  |  | 2. Is a pediatric hematology-oncology physician part of the primary medical team responsible for the care of critically ill PHO patients? (Provides daily care and participates in daily rounds) | a) Always (100% of the time or 24 hrs. a day/7 day a week) | 5 |  |
|  |  |  |  | b) Almost Always (80-99% of the time or 24 hrs. Monday-Friday and some hours on the weekends) | 4 |  |
|  |  |  |  | c) Frequently (60-79% of the time or 24 hrs. Monday-Friday) | 3 |  |
|  |  |  |  | d) Sometimes/Moderate availability (41-59% of the time or only daytime 5-7 days a week) | 2 |  |
|  |  |  |  | e) Infrequently/Limited availability (21-40% of the time or only daytime 3-4 days a week) | 1 |  |
|  |  |  |  | f) Almost Never (1-20% of the time or only daytime 1-2 days a week) | 0 |  |
|  |  |  |  | g) Never/Not available | 0 |  |

| Personnel | Core Team | PICU | 3. Is nursing staff formally trained in pediatric critical care part of the primary medical team responsible for the care of critically ill PHO patients? (trained nurse is actively involved in daily care of patients and not merely a resource for non-ICU nurses) | a) Always (100% of the time or 24 hrs. a day/7 day a week) | 5 |  |
| --- | --- | --- | --- | --- | --- | --- |
|  |  |  |  | b) Almost Always (80-99% of the time or 24 hrs. Monday-Friday and some hours on the weekends) | 4 |  |
|  |  |  |  | c) Frequently (60-79% of the time or 24 hrs. Monday-Friday) | 3 |  |
|  |  |  |  | d) Sometimes/Moderate availability (41-59% of the time or only daytime 5-7 days a week) | 2 |  |
|  |  |  |  | e) Infrequently/Limited availability (21-40% of the time or only daytime 3-4 days a week) | 1 |  |
|  |  |  |  | f) Almost Never (1-20% of the time or only daytime 1-2 days a week) | 0 |  |
|  |  |  |  | g) Never/Not available | 0 |  |
|  |  |  | 3a. What is the total number of pediatric critical care nurses working in the PICU (or area where critically ill PHO patients are treated) in your hospital? (Formally trained in PCCM) | a)  ______ (number) | N/A |  |
|  |  |  | 3b. Are nurses with experience managing critically ill PHO patients part of the primary medical team? | a) Always (100% of the time or 24 hrs. a day/7 day a week) | 5 |  |
|  |  |  |  | b) Almost Always (80-99% of the time or 24 hrs. Monday-Friday and some hours on the weekends) | 4 |  |
|  |  |  |  | c) Frequently (60-79% of the time or 24 hrs. Monday-Friday) | 3 |  |
|  |  |  |  | d) Sometimes/Moderate availability (41-59% of the time or only daytime 5-7 days a week) | 2 |  |
|  |  |  |  | e) Infrequently/Limited availability (21-40% of the time or only daytime 3-4 days a week) | 1 |  |
|  |  |  |  | f) Almost Never (1-20% of the time or only daytime 1-2 days a week) | 0 |  |
|  |  |  |  | g) Never/Not available | 0 |  |
|  | Core Consultants | PICU | 1. How often are pediatric critical care physician available for in-person consults on critically ill PHO patients? (Some consults can be done by phone, but consultants examine patients within 24 hrs. after being consulted) | a)  Always (100% of the time or 24 hrs. a day/7 day a week) | 5 |  |
|  |  |  |  | b) Almost Always (80-99% of the time or 24 hrs. Monday-Friday and some hours on the weekends) | 4 |  |
|  |  |  |  | c) Frequently (60-79% of the time or 24 hrs. Monday-Friday) | 3 |  |
|  |  |  |  | d) Sometimes/Moderate availability (41-59% of the time or only daytime 5-7 days a week) | 2 |  |
|  |  |  |  | e) Infrequently/Limited availability (21-40% of the time or only daytime 3-4 days a week) | 1 |  |
|  |  |  |  | f)  Almost Never (1-20% of the time or only daytime 1-2 days a week) | 0 |  |
|  |  |  |  | g) Never/Not available | 0 |  |
|  |  |  | 2. How often are pediatric hematology-oncology physician available for in-person consults on critically ill PHO patients? (Some consults can be done by phone, but consultants examine patients within 24 hrs. after being consulted) | a) Always (100% of the time or 24 hrs. a day/7 day a week) | 5 |  |
|  |  |  |  | b) Almost Always (80-99% of the time or 24 hrs. Monday-Friday and some hours on the weekends) | 4 |  |
|  |  |  |  | c) Frequently (60-79% of the time or 24 hrs. Monday-Friday) | 3 |  |
|  |  |  |  | d) Sometimes/Moderate availability (41-59% of the time or only daytime 5-7 days a week) | 2 |  |
|  |  |  |  | e) Infrequently/Limited availability (21-40% of the time or only daytime 3-4 days a week) | 1 |  |
|  |  |  |  | f)  Almost Never (1-20% of the time or only daytime 1-2 days a week) | 0 |  |
|  |  |  |  | g) Never/Not available | 0 |  |
|  |  |  | 3. How often are pediatric surgeons available for in-person consults on critically ill PHO patients? (Some consults can be done by phone, but consultants examine patients within 24 hrs. after being consulted) | a) Always (100% of the time or 24 hrs. a day/7 day a week) | 5 |  |
|  |  |  |  | b) Almost Always (80-99% of the time or 24 hrs. Monday-Friday and some hours on the weekends) | 4 |  |
|  |  |  |  | c) Frequently (60-79% of the time or 24 hrs. Monday-Friday) | 3 |  |
|  |  |  |  | d) Sometimes/Moderate availability (41-59% of the time or only daytime 5-7 days a week) | 2 |  |
|  |  |  |  | e) Infrequently/Limited availability (21-40% of the time or only daytime 3-4 days a week) | 1 |  |
|  |  |  |  | f) Almost Never (1-20% of the time or only daytime 1-2 days a week) | 0 |  |
|  |  |  |  | g) Never/Not available | 0 |  |
|  |  |  | 4. How often are pediatric neurosurgeons available for in-person consults on critically ill PHO patients? (Some consults can be done by phone, but consultants examine patients within 24 hrs. after being consulted) | a) Always (100% of the time or 24 hrs. a day/7 day a week) | 5 |  |
|  |  |  |  | b) Almost Always (80-99% of the time or 24 hrs. Monday-Friday and some hours on the weekends) | 4 |  |
|  |  |  |  | c) Frequently (60-79% of the time or 24 hrs. Monday-Friday) | 3 |  |
|  |  |  |  | d) Sometimes/Moderate availability (41-59% of the time or only daytime 5-7 days a week) | 2 |  |
|  |  |  |  | e) Infrequently/Limited availability (21-40% of the time or only daytime 3-4 days a week) | 1 |  |
|  |  |  |  | f) Almost Never (1-20% of the time or only daytime 1-2 days a week) | 0 |  |
|  |  |  |  | g) No pediatric intensivist available | 0 |  |
|  | Staffing | PICU | 1. How often is a pediatric critical care physician (Not fellows) in the hospital staffing the PICU (or area where critically ill PHO patients are treated) in your hospital? | a) Always (100% of the time or 24 hrs. a day/7 day a week) | 5 |  |
|  |  |  |  | b) Almost Always (80-99% of the time or 24 hrs. Monday-Friday and some hours on the weekends) | 4 |  |
|  |  |  |  | c) Frequently (60-79% of the time or 24 hrs. Monday-Friday) | 3 |  |
|  |  |  |  | d) Sometimes/Moderate availability (41-59% of the time or only daytime 5-7 days a week) | 2 |  |

| Personnel | Staffing | PICU |  | e) Infrequently/Limited availability (21-40% of the time or only daytime 3-4 days a week) | 1 |  |
| --- | --- | --- | --- | --- | --- | --- |
|  |  |  |  | f) Almost Never (1-20% of the time or only daytime 1-2 days a week) | 0 |  |
|  |  |  |  | g) Never/Not available | 0 |  |
|  |  |  | If the answer is G for question 1 (NO pediatric intensivist available):  1a. How often is a pediatrician with experience managing critically ill PHO staffing the PICU (or area where critically ill PHO patients are treated) in your hospital? | a) Always (100% of the time or 24 hrs. a day/7 day a week) | 5 |  |
|  |  |  |  | b) Almost Always (80-99% of the time or 24 hrs. Monday-Friday and some hours on the weekends) | 4 |  |
|  |  |  |  | c) Frequently (60-79% of the time or 24 hrs. Monday-Friday) | 3 |  |
|  |  |  |  | d) Sometimes/Moderate availability (41-59% of the time or only daytime 5-7 days a week) | 2 |  |
|  |  |  |  | e) Infrequently/Limited availability (21-40% of the time or only daytime 3-4 days a week) | 1 |  |
|  |  |  |  | f) Almost Never (1-20% of the time or only daytime 1-2 days a week) | 0 |  |
|  |  |  |  | g) Another physician (e.g., oncologist), please describe: | 0 |  |
|  |  |  | 2. What was the nurse-to-patient ratio in the PICU (or area where critically ill PHO patients are treated) during day-time shifts, 7 days a week PRIOR to the COVID-19 pandemic? | a) 1 nurse : 1-2 patients | 5 |  |
|  |  |  |  | b) 1 nurse : 3-4 patients | 3 |  |
|  |  |  |  | c) 1 nurse : 5-6 patients | 1 |  |
|  |  |  |  | d) 1 nurse : 7 or more patients, please describe: | 0 |  |
|  |  |  | 3. What is the current nurse-to-patient ratio in the PICU (or area where critically ill PHO patients are treated) during day-time shifts, 7 days a week? (During the COVID-19 pandemic) | a) 1 nurse : 1-2 patients | 5 |  |
|  |  |  |  | b) 1 nurse : 3-4 patients | 3 |  |
|  |  |  |  | c) 1 nurse : 5-6 patients | 1 |  |
|  |  |  |  | d) 1 nurse : 7 or more patients, please describe: | 0 |  |
|  |  |  | 4. What was the nurse-to-patient ratio in the PICU (or area where critically ill PHO patients are treated) during night-time shifts, 7 days a week PRIOR to the COVID-19 pandemic? | a) 1 nurse : 1-2 patients | 5 |  |
|  |  |  |  | b) 1 nurse : 3-4 patients | 3 |  |
|  |  |  |  | c) 1 nurse : 5-6 patients | 1 |  |
|  |  |  |  | d) 1 nurse : 7 or more patients, please describe: | 0 |  |
|  |  |  | 5. What is the current nurse-to-patient ratio in the PICU (or area where critically ill PHO patients are treated) during night-time shifts, 7 days a week? (During the COVID-19 pandemic) | a) 1 nurse : 1-2 patients | 5 |  |
|  |  |  |  | b) 1 nurse : 3-4 patients | 3 |  |
|  |  |  |  | c) 1 nurse : 5-6 patients | 1 |  |
|  |  |  |  | d) 1 nurse : 7 or more patients, please describe: | 0 |  |
|  |  |  | 6. How often is there a charge nurse/nursing supervisor responsible for the organization, management, supervision and quality of nursing practices in the PICU (or area where critically ill PHO patients are treated) available during each shift (day and night)? | a) Always (100% of the time or 24 hrs. a day/7 day a week) | 5 |  |
|  |  |  |  | b) Almost Always (80-99% of the time or 24 hrs. Monday-Friday and some hours on the weekends) | 4 |  |
|  |  |  |  | c) Frequently (60-79% of the time or 24 hrs. Monday-Friday) | 3 |  |
|  |  |  |  | d) Sometimes/Moderate availability (41-59% of the time or only daytime 5-7 days a week) | 2 |  |
|  |  |  |  | e) Infrequently/Limited availability (21-40% of the time or only daytime 3-4 days a week) | 1 |  |
|  |  |  |  | f) Almost Never (1-20% of the time or only daytime 1-2 days a week) | 0 |  |
|  |  |  |  | g) Never/Not available | 0 |  |
|  | Education, Research and QI | PICU | 1. Does your hospital have a formal orientation program focused on pediatric critical care for newly hired nurses in the PICU? | a) Yes | 5 |  |
|  |  |  |  | b) No | 0 |  |
|  |  |  | 2. Does your hospital have on-line or on-site educator or workshops available for the professional development, training and education of pediatric critical care nurses? | a) Yes | 5 |  |
|  |  |  |  | b) No | 0 |  |
|  |  |  | 3. What is the percentage of physicians caring for critically ill pediatric patients (including critically ill PHO patients) who have up-to-date (not expired) pediatric cardiorespiratory resuscitation certification (e.g., PALS, BLS)? | a) 80-100% | 5 |  |
|  |  |  |  | b) 60-79% | 3 |  |
|  |  |  |  | c) 40-59% | 2 |  |
|  |  |  |  | d) 20-39% | 1 |  |
|  |  |  |  | e) 1-19% | 0 |  |
|  |  |  |  | f)  Do not know | 0 |  |
|  |  |  | 4. What is the percentage of nurses caring for critically ill pediatric patients (including critically ill PHO patients) who have up-to-date (not expired) pediatric cardiorespiratory resuscitation certification (e.g., PALS, BLS)? | a) 80-100% | 5 |  |
|  |  |  |  | b) 60-79% | 3 |  |
|  |  |  |  | c) 40-59% | 2 |  |
|  |  |  |  | d) 20-39% | 1 |  |

| Personnel | Education, Research and QI | PICU |  | e) 1-19% | 0 |  |
| --- | --- | --- | --- | --- | --- | --- |
|  |  |  |  | f) Do not know | 0 |  |
|  |  |  | 5. How often do medical trainees (fellows and residents), physician assistants and nurse practitioners work under the direct (in-person) supervision of an attending physician when caring for critically ill PHO patient in the PICU? | a) Always (24 hrs. a day/7 day a week or 100% of the time) | 5 |  |
|  |  |  |  | b) Almost Always (80-99% of the time) | 4 |  |
|  |  |  |  | c) Frequently (60-79% of the time) | 3 |  |
|  |  |  |  | d) Sometimes/Moderate availability (41-59% of the time) | 2 |  |
|  |  |  |  | e) Infrequently/Limited availability (21-40% of the time) | 1 |  |
|  |  |  |  | f) Almost Never (1-20% of the time) | 0 |  |
|  |  |  |  | g) No direct supervision available | 0 |  |
|  |  |  | 6. Is there free access to resources for reviewing the latest medical literature for healthcare providers (e.g., PubMed, UpToDate, hard copies of journals) at your hospital? | a) Yes | 5 |  |
|  |  |  |  | b) No | 0 |  |
|  |  |  | 7.  Does the pediatric critical care team participate in research or quality improvement projects designed to improve the care of critically ill PHO patients? | a) Yes | 5 |  |
|  |  |  |  | b)  No | 0 |  |
|  |  |  | If the answer is Yes free access for question 7, please check all that apply: i. Education ii. Quality improvement or patient care delivery iii. Research iv. Other, please explain: | | |  |
| Service Capacity | Service Organization | PICU | 1. Does your hospital have a “closed” PICU system, where the primary responsibility and management of admitted critically ill PHO patients is transferred to the pediatric critical care physician or pediatrician in charge? | a) Yes | 5 |  |
|  |  |  |  | b) No, please describe: | 0 |  |
|  |  |  | 2. How often is an attending physician (not trainees) primarily responsible for the medical decisions involving critically ill PHO patients? (If trainees or other healthcare providers make the decisions, these are approved by an attending physician) | a) Always (100% of the time or 24 hrs. a day/7 day a week) | 5 |  |
|  |  |  |  | b) Almost Always (80-99% of the time) | 4 |  |
|  |  |  |  | c) Frequently (60-79% of the time) | 3 |  |
|  |  |  |  | d) Sometimes (41-59% of the time) | 2 |  |
|  |  |  |  | e) Infrequently (21-40% of the time) | 1 |  |
|  |  |  |  | f) Almost Never (1-20% of the time) | 0 |  |
|  |  |  |  | g) Never/Not available | 0 |  |
|  |  |  | 3. How often is a healthcare provider trained in performing procedures, including but not limited to endotracheal intubation, arterial line, central line, and chest tube placement, available as needed for the care of critically ill PHO? | a) Always (100% of the time or 24 hrs. a day/7 day a week) | 5 |  |
|  |  |  |  | b) Almost Always (80-99% of the time) | 4 |  |
|  |  |  |  | c) Frequently (60-79% of the time) | 3 |  |
|  |  |  |  | d) Sometimes/Moderate availability (41-59% of the time) | 2 |  |
|  |  |  |  | e) Infrequently/Limited availability (21-40% of the time) | 1 |  |
|  |  |  |  | f) Almost Never (1-20% of the time) | 0 |  |
|  |  |  |  | g) Never/Not available | 0 |  |
|  | Acute Management of Critically ill PHO patients | PICU | 1. Is there a system (operational plan) in place to manage critically ill PHO patients in the Emergency Department (ED) or wards, if NO beds are available in the PICU? | a) Yes | 5 |  |
|  |  |  |  | b) No | 0 |  |
|  |  |  | 2. Is there a system (operational plan) in place to divert or transfer critically ill PHO patients to other hospitals if NO beds are available in the PICU? | a) Yes | 5 |  |
|  |  |  |  | b) No | 0 |  |
|  |  |  | 3. How often are hospitalized PHO patients who require escalation of care transferred in a timely manner (within 4 hours) to the PICU (or the area where critically ill patients are treated) from other in-patient hospital units (e.g., PHO unit)? | a) Always (100% of the time) | 5 |  |
|  |  |  |  | b) Almost Always (80-99% of the time) | 4 |  |
|  |  |  |  | c) Frequently (60-79% of the time) | 3 |  |
|  |  |  |  | d) Sometimes (41-59% of the time) | 2 |  |
|  |  |  |  | e) Infrequently (21-40% of the time) | 1 |  |
|  |  |  |  | f) Almost Never (1-20% of the time) | 0 |  |
|  |  |  |  | g) No PICU or area for critically ill pediatric patients available | 0 |  |
|  |  |  | 3a. How long does it typically take to transfer patients from the inpatient units to the PICU or areas where critically ill patients are treated? | a) < 1 hr. | 5 |  |
|  |  |  |  | b) 1-3 hrs. | 3 |  |
|  |  |  |  | c) 4-8 hrs. | 1 |  |
|  |  |  |  | d) > 8 hrs. | 0 |  |

| Service Capacity | Acute Management of Critically ill PHO patients | ONC | 1. How often are vasopressors or inotropes used on the floor/ward/inpatient care area (rather than in a PICU or area where critically ill PHO patients are treated) for hospitalized PHO patients requiring vasoactive infusions, due to lack of ICU beds? | a) Always (100% of the time) | 0 |  |
| --- | --- | --- | --- | --- | --- | --- |
|  |  |  |  | b) Almost Always (80-99% of the time) | 0 |  |
|  |  |  |  | c) Frequently (60-79% of the time) | 1 |  |
|  |  |  |  | d) Sometimes (41-59% of the time) | 2 |  |
|  |  |  |  | e) Infrequently (21-40% of the time) | 3 |  |
|  |  |  |  | f) Almost Never (1-20% of the time) | 4 |  |
|  |  |  |  | g) Never | 5 |  |
|  |  |  | 2. How often is invasive mechanical ventilation used on the floor/ward/ inpatient care area (rather than in a PICU or area where critically ill PHO patients are treated) for hospitalized PHO patients with respiratory failure due to lack of ICU beds? | a) Always (100% of the time) | 0 |  |
|  |  |  |  | b) Almost Always (80-99% of the time) | 0 |  |
|  |  |  |  | c) Frequently (60-79% of the time) | 1 |  |
|  |  |  |  | d) Sometimes (41-59% of the time) | 2 |  |
|  |  |  |  | e) Infrequently (21-40% of the time) | 3 |  |
|  |  |  |  | f) Almost Never (1-20% of the time) | 4 |  |
|  |  |  |  | g) Never | 5 |  |
|  |  |  | 3. How often are hospitalized PHO patients who develop critical illness (altered mental status, renal failure requiring dialysis, respiratory insufficiency, etc.) managed on the floor/ward/inpatient care area (rather than in a PICU or area where critically ill PHO patients are treated) due to lack of ICU beds? | a) Always (100% of the time) | 0 |  |
|  |  |  |  | b) Almost Always (80-99% of the time) | 0 |  |
|  |  |  |  | c) Frequently (60-79% of the time) | 1 |  |
|  |  |  |  | d) Sometimes (41-59% of the time) | 2 |  |
|  |  |  |  | e) Infrequently (21-40% of the time) | 3 |  |
|  |  |  |  | f) Almost Never (1-20% of the time) | 4 |  |
|  |  |  |  | g) Never | 5 |  |
|  | Standard Precautions and Infection Prevention in the intensive care | PICU | 1. How often are Examination Gloves available for use in the management of critically ill PHO patients? | a) Always (100% of the time) | 5 |  |
|  |  |  |  | b) Almost Always (80-99% of the time) | 4 |  |
|  |  |  |  | c) Frequently (60-79% of the time) | 3 |  |
|  |  |  |  | d) Sometimes/Moderate availability (41-59% of the time) | 2 |  |
|  |  |  |  | e) Infrequently/Limited availability (21-40% of the time) | 1 |  |
|  |  |  |  | f) Almost Never (1-20% of the time) | 0 |  |
|  |  |  |  | g) Never/Not available | 0 |  |
|  |  |  | 2. How often are Isolation gowns available for use in the management of critically ill PHO patients? | a) Always (100% of the time) | 5 |  |
|  |  |  |  | b) Almost Always (80-99% of the time) | 4 |  |
|  |  |  |  | c) Frequently (60-79% of the time) | 3 |  |
|  |  |  |  | d) Sometimes/Moderate availability (41-59% of the time) | 2 |  |
|  |  |  |  | e) Infrequently/Limited availability (21-40% of the time) | 1 |  |
|  |  |  |  | f) Almost Never (1-20% of the time) | 0 |  |
|  |  |  |  | g) Never/Not available | 0 |  |
|  |  |  | 3. How often are Masks (e.g., surgical mask, N95) available for use in the management of critically ill PHO patients? | a) Always (100% of the time) | 5 |  |
|  |  |  |  | b) Almost Always (80-99% of the time) | 4 |  |
|  |  |  |  | c) Frequently (60-79% of the time) | 3 |  |
|  |  |  |  | d) Sometimes/Moderate availability (41-59% of the time) | 2 |  |
|  |  |  |  | e) Infrequently/Limited availability (21-40% of the time) | 1 |  |
|  |  |  |  | f) Almost Never (1-20% of the time) | 0 |  |
|  |  |  |  | g) Never/Not available | 0 |  |
|  |  |  | 4 How often are Eye shields or protective goggles available for use in the management of critically ill PHO patients? | a) Always (100% of the time) | 5 |  |
|  |  |  |  | b) Almost Always (80-99% of the time) | 4 |  |
|  |  |  |  | c) Frequently (60-79% of the time) | 3 |  |
|  |  |  |  | d) Sometimes/Moderate availability (41-59% of the time) | 2 |  |
|  |  |  |  | e) Infrequently/Limited availability (21-40% of the time) | 1 |  |
|  |  |  |  | f) Almost Never (1-20% of the time) | 0 |  |
|  |  |  |  | g) Never/Not available | 0 |  |

| Service Capacity | Standard Precautions and Infection Prevention in the intensive care | PICU | 5. How often are Shoe covers available for use in the management of critically ill PHO patients? | a) Always (100% of the time) | 5 |  |
| --- | --- | --- | --- | --- | --- | --- |
|  |  |  |  | b) Almost Always (80-99% of the time) | 4 |  |
|  |  |  |  | c) Frequently (60-79% of the time) | 3 |  |
|  |  |  |  | d) Sometimes/Moderate availability (41-59% of the time) | 2 |  |
|  |  |  |  | e) Infrequently/Limited availability (21-40% of the time) | 1 |  |
|  |  |  |  | f)  Almost Never (1-20% of the time) | 0 |  |
|  |  |  |  | g) Never/Not available | 0 |  |
|  |  |  | 6. How often are head covers or caps available for use in the management of critically ill PHO patients? | a) Always (100% of the time) | 5 |  |
|  |  |  |  | b) Almost Always (80-99% of the time) | 4 |  |
|  |  |  |  | c) Frequently (60-79% of the time) | 3 |  |
|  |  |  |  | d) Sometimes/Moderate availability (41-59% of the time) | 2 |  |
|  |  |  |  | e) Infrequently/Limited availability (21-40% of the time) | 1 |  |
|  |  |  |  | f) Almost Never (1-20% of the time) | 0 |  |
|  |  |  |  | g) Never/Not available | 0 |  |
|  |  |  | 7. How often are Sterile Gloves regularly available to perform procedures (e.g., line placement) on critically ill PHO patients? | a) Always (100% of the time) | 5 |  |
|  |  |  |  | b) Almost Always (80-99% of the time) | 4 |  |
|  |  |  |  | c) Frequently (60-79% of the time) | 3 |  |
|  |  |  |  | d) Sometimes/Moderate availability (41-59% of the time) | 2 |  |
|  |  |  |  | e) Infrequently/Limited availability (21-40% of the time) | 1 |  |
|  |  |  |  | f) Almost Never (1-20% of the time) | 0 |  |
|  |  |  |  | g) Never/Not available | 0 |  |
|  |  |  | 8. How often are Sterile Gowns regularly available to perform procedures (e.g., line placement) on critically ill PHO patients? | a) Always (100% of the time) | 5 |  |
|  |  |  |  | b) Almost Always (80-99% of the time) | 4 |  |
|  |  |  |  | c) Frequently (60-79% of the time) | 3 |  |
|  |  |  |  | d) Sometimes/Moderate availability (41-59% of the time) | 2 |  |
|  |  |  |  | e) Infrequently/Limited availability (21-40% of the time) | 1 |  |
|  |  |  |  | f) Almost Never (1-20% of the time) | 0 |  |
|  |  |  |  | g) Never/Not available | 0 |  |
|  |  |  | 9. How often is there adequate access to hand-washing sinks equipped with soap and paper towels near the entrance to each critically ill PHO patient’s room in the PICU (or area where critically ill PHO patients are treated)? | a) Always (100% of the time) | 5 |  |
|  |  |  |  | b) Almost Always (80-99% of the time) | 4 |  |
|  |  |  |  | c) Frequently (60-79% of the time) | 3 |  |
|  |  |  |  | d) Sometimes/Moderate availability (41-59% of the time) | 2 |  |
|  |  |  |  | e) Infrequently/Limited availability (21-40% of the time) | 1 |  |
|  |  |  |  | f) Almost Never (1-20% of the time) | 0 |  |
|  |  |  |  | g) Never/Not available | 0 |  |
|  |  |  | 10. How often is there adequate access to functional and stocked alcohol-based hand sanitizer dispensers by the bed of each critically ill PHO patient in the PICU (or area where critically ill PHO patients are treated)? | a) Always (100% of the time) | 5 |  |
|  |  |  |  | b) Almost Always (80-99% of the time) | 4 |  |
|  |  |  |  | c) Frequently (60-79% of the time) | 3 |  |
|  |  |  |  | d) Sometimes/Moderate availability (41-59% of the time) | 2 |  |
|  |  |  |  | e) Infrequently/Limited availability (21-40% of the time) | 1 |  |
|  |  |  |  | f) Almost Never (1-20% of the time) | 0 |  |
|  |  |  |  | g) Never/Not available | 0 |  |
|  |  |  | 11. Is there a standardized process to monitor adherence to hand hygiene protocols by healthcare personnel at your hospital? | a) Yes | 5 |  |
|  |  |  |  | b) No | 0 |  |

| Service Capacity | Pediatric Outreach Team | ONC | 1. Does your hospital have a system in place to aid in the observation, monitoring and escalation of care (e.g., PEWS—Pediatric Early Warning System) for hospitalized PHO patients? | a) Yes | 5 |  |
| --- | --- | --- | --- | --- | --- | --- |
|  |  |  |  | b) No | 0 |  |
|  |  |  |  | c) Do not know | 0 |  |
|  |  |  | 2. How often is a pediatric critical care physician or trained healthcare provider (critical care outreach team) available for urgent in-person consultation (within 30 minutes) on hospitalized PHO patients presenting with acute clinical changes or deterioration? | a) Always (24 hrs. a day/ 7 days a week or 100% of the time) | 5 |  |
|  |  |  |  | b) Almost Always (80-99% of the time) | 4 |  |
|  |  |  |  | c) Frequently (60-79% of the time) | 3 |  |
|  |  |  |  | d) Sometimes/Moderate availability (41-59% of the time) | 2 |  |
|  |  |  |  | e) Infrequently/Limited availability (21-40% of the time) | 1 |  |
|  |  |  |  | f) Almost Never (1-20% of the time) | 0 |  |
|  |  |  |  | g) Never/Not available | 0 |  |
|  |  |  | 3. How often is a pediatric rapid response team available (within 10-15 minutes) for hospitalized PHO patients who develop signs of acute clinical deterioration? | a) Always (24 hrs. a day/ 7 days a week or 100% of the time) | 5 |  |
|  |  |  |  | b) Almost Always (80-99% of the time) | 4 |  |
|  |  |  |  | c) Frequently (60-79% of the time) | 3 |  |
|  |  |  |  | d) Sometimes/Moderate availability (41-59% of the time) | 2 |  |
|  |  |  |  | e) Infrequently/Limited availability (21-40% of the time) | 1 |  |
|  |  |  |  | f) Almost Never (1-20% of the time) | 0 |  |
|  |  |  |  | g) Never/Not available | 0 |  |
|  |  |  | 4. How often is a pediatric code blue resuscitation team available (within 5 minutes) for hospitalized PHO patients with signs of cardiorespiratory arrest? | a) Always (24 hrs. a day/ 7 days a week or 100% of the time) | 5 |  |
|  |  |  |  | b) Almost Always (80-99% of the time) | 4 |  |
|  |  |  |  | c) Frequently (60-79% of the time) | 3 |  |
|  |  |  |  | d) Sometimes/Moderate availability (41-59% of the time) | 2 |  |
|  |  |  |  | e) Infrequently/Limited availability (21-40% of the time) | 1 |  |
|  |  |  |  | f) Almost Never (1-20% of the time) | 0 |  |
|  |  |  |  | g) Never/Not available | 0 |  |
|  |  |  | 5. How often is pediatric-size equipment (e.g., paddles, endotracheal tubes) easily accessible in a centralized location (“crash cart”) when needed for acute resuscitation of critically ill pediatric patients (including critically ill PHO patients) at your hospital? | a) Always (24 hrs. a day/ 7 days a week or 100% of the time) | 5 |  |
|  |  |  |  | b) Almost Always (80-99% of the time) | 4 |  |
|  |  |  |  | c) Frequently (60-79% of the time) | 3 |  |
|  |  |  |  | d) Sometimes/Moderate availability (41-59% of the time) | 2 |  |
|  |  |  |  | e) Infrequently/Limited availability (21-40% of the time) | 1 |  |
|  |  |  |  | f) Almost Never (1-20% of the time) | 0 |  |
|  |  |  |  | g) Never/Not available | 0 |  |
|  |  |  | 6. How often does your hospital perform regular maintenance “crash cart” inventory to confirm accessibility to appropriate equipment and emergency drugs? (Daily check for missing drugs and at least monthly for expired medications and batteries on the defibrillator or AED) | a) Always (100% of the time) | 5 |  |
|  |  |  |  | b) Almost Always (80-99% of the time) | 4 |  |
|  |  |  |  | c) Frequently (60-79% of the time) | 3 |  |
|  |  |  |  | d) Sometimes (41-59% of the time) | 2 |  |
|  |  |  |  | e) Infrequently (21-40% of the time) | 1 |  |
|  |  |  |  | f) Almost Never (1-20% of the time) | 0 |  |
|  |  |  |  | g) Never/Not available | 0 |  |
|  | Guidelines and Protocols | PICU | 1. How often are newly admitted critically ill PHO patients assessed (within 5 min) upon arrival to the PICU (or area where critically ill PHO patients are treated) by a physician or trained healthcare provider? | a) Always (100% of the time) | 5 |  |
|  |  |  |  | b) Almost Always (80-99% of the time) | 4 |  |
|  |  |  |  | c) Frequently (60-79% of the time) | 3 |  |
|  |  |  |  | d) Sometimes (41-59% of the time) | 2 |  |
|  |  |  |  | e) Infrequently (21-40% of the time) | 1 |  |
|  |  |  |  | f) Almost Never (1-20% of the time) | 0 |  |
|  |  |  |  | g) Never | 0 |  |
|  |  |  | 2. Does your hospital have a standardized process to confirm the identity of critically ill PHO patients (e.g., unique patient identifiers) prior to the administration of medications (e.g., chemotherapy) and/or procedures? | a) Yes | 5 |  |
|  |  |  |  | b) No | 0 |  |

| Service Capacity | Guidelines and Protocols | PICU | 3. Is there a clinical guideline (updated with the latest medical evidence) for the management and treatment of sepsis/septic shock in critically ill PHO patients? | a) Yes, and the guideline has been updated with relevant published science in the last 5 years | 5 |  |
| --- | --- | --- | --- | --- | --- | --- |
|  |  |  |  | b) Yes, but the guideline has NOT been updated with relevant published science in ≥ 5 years | 3 |  |
|  |  |  |  | c)  No clinical guideline available | 0 |  |
|  |  |  |  | d) Do not know | 0 |  |
|  |  |  | 4. Is there a clinical guideline (updated with the latest medical evidence) for the management and treatment of seizure and status epilepticus in critically ill pediatric patients (including critically ill PHO patients)? | a) Yes, and the guideline has been updated with relevant published science in the last 5 years | 5 |  |
|  |  |  |  | b) Yes, but the guideline has NOT been updated with relevant published science in ≥ 5 years | 3 |  |
|  |  |  |  | c) No clinical guideline available | 0 |  |
|  |  |  |  | d) Do not know | 0 |  |
|  |  |  | 5. Is there a clinical guideline (updated with the latest medical evidence) for the management and treatment of increased intracranial pressure in critically ill pediatric patients (including critically ill PHO patients)? | a) Yes, and the guideline has been updated with relevant published science in the last 5 years | 5 |  |
|  |  |  |  | b) Yes, but the guideline has NOT been updated with relevant published science in ≥ 5 years | 3 |  |
|  |  |  |  | c) No clinical guideline available | 0 |  |
|  |  |  |  | d) Do not know | 0 |  |
|  |  |  | 6. Is there a clinical guideline (updated with the latest medical evidence) for the management and treatment of respiratory failure in critically ill PHO patients? | a) Yes, and the guideline has been updated with relevant published science in the last 5 years | 5 |  |
|  |  |  |  | b) Yes, but the guideline has NOT been updated with relevant published science in ≥ 5 years | 3 |  |
|  |  |  |  | c) No clinical guideline available | 0 |  |
|  |  |  |  | d) Do not know | 0 |  |
|  |  |  | 7. Is there a clinical guideline (updated with the latest medical evidence) for the management and treatment of blood products transfusion reactions or transfusion related complications in critically ill PHO patients? | a) Yes, and the guideline has been updated with relevant published science in the last 5 years | 5 |  |
|  |  |  |  | b) Yes, but the guideline has NOT been updated with relevant published science in ≥ 5 years | 3 |  |
|  |  |  |  | c) No clinical guideline available | 0 |  |
|  |  |  |  | d) Do not know | 0 |  |
|  |  |  | 8. Is there a standardized process for the transport of critically ill PHO patients (within the hospital, to the PICU from other facilities, and from the PICU to other facilities)? | a) Yes | 5 |  |
|  |  |  |  | b) No | 0 |  |
|  |  |  | 9. Is there a standardized process to monitor adherence to hospital policies and clinical guidelines for critically ill pediatric patient at your hospital? | a) Yes | 5 |  |
|  |  |  |  | b) No | 0 |  |
|  |  | ONC | 1. Is there a clinical guideline (updated with the latest medical evidence) for the management and treatment of febrile neutropenia in critically ill PHO patients? | a) Yes, and the guideline has been updated with relevant published science in the last 5 years | 5 |  |
|  |  |  |  | b) Yes, but the guideline has NOT been updated with relevant published science in ≥ 5 years | 3 |  |
|  |  |  |  | c) No clinical guideline available | 0 |  |
|  |  |  |  | d) Do not know | 0 |  |
|  |  |  | 2. Is there a clinical guideline (updated with the latest medical evidence) for the management and treatment of tumor lysis syndrome in critically ill PHO patients? | a) Yes, and the guideline has been updated with relevant published science in the last 5 years | 5 |  |
|  |  |  |  | b) Yes, but the guideline has NOT been updated with relevant published science in ≥ 5 years | 3 |  |
|  |  |  |  | c) No clinical guideline available | 0 |  |
|  |  |  |  | d) Do not know | 0 |  |
|  |  |  | 3. Is there a clinical guideline (updated with the latest medical evidence) for the management and treatment of chemotherapy related toxicities (e.g., hemorrhagic cystitis, methotrexate toxicity) in critically ill PHO patients? | a) Yes, and the guideline has been updated with relevant published science in the last 5 years | 5 |  |
|  |  |  |  | b) Yes, but the guideline has NOT been updated with relevant published science in ≥ 5 years | 3 |  |
|  |  |  |  | c) No clinical guideline available | 0 |  |
|  |  |  |  | d) Do not know | 0 |  |
|  |  |  | 4. Is there a policy that allows families to request a medical order for DNR allowing the medical team to withhold CPR of a dying pediatric patient at your hospital? | a) Yes | 5 |  |
|  |  |  |  | b) No | 0 |  |
|  |  |  | 5. Are there standardized updated evidence-based guidelines, protocols, or checklists to guide the clinical care of critically ill PHO patients at your hospital (e.g., pain)? | a) Yes | 5 |  |
|  |  |  |  | b) No | 0 |  |
| Service Integration | Communication  and  Multidisciplinary  Care | PICU | 1. How often is there daily documented communication between the PICU/IMCU/HDU and the hematology/oncology team regarding management and treatment of critically ill PHO patients? | a) Always (100% of the time) | 5 |  |
|  |  |  |  | b) Almost Always (80-99% of the time) | 4 |  |
|  |  |  |  | c) Frequently (60-79% of the time) | 3 |  |
|  |  |  |  | d) Sometimes (41-59% of the time) | 2 |  |
|  |  |  |  | e) Infrequently (21-40% of the time) | 1 |  |
|  |  |  |  | f) Almost Never (1-20% of the time) | 0 |  |
|  |  |  |  | g) No communication documented | 0 |  |

| Service Integration | Communication  and  Multidisciplinary  Care | PICU | 2. How often is daily documented communication occurring between the PICU/IMCU/HDU and the surgical team regarding surgical procedures and post operatively management of critically ill PHO patients requiring surgical care (pre/post-op)? | a) Always (100% of the time) | 5 |  |
| --- | --- | --- | --- | --- | --- | --- |
|  |  |  |  | b) Almost Always (80-99% of the time) | 4 |  |
|  |  |  |  | c) Frequently (60-79% of the time) | 3 |  |
|  |  |  |  | d) Sometimes (41-59% of the time) | 2 |  |
|  |  |  |  | e) Infrequently (21-40% of the time) | 1 |  |
|  |  |  |  | f) Almost Never (1-20% of the time) | 0 |  |
|  |  |  |  | g) No communication documented | 0 |  |
|  |  |  | 3. How often are daily multidisciplinary patient care rounds conducted to discuss the management of critically ill PHO patients? | a) Always (100% of the time) | 5 |  |
|  |  |  |  | b) Almost Always (80-99% of the time) | 4 |  |
|  |  |  |  | c) Frequently (60-79% of the time) | 3 |  |
|  |  |  |  | d) Sometimes (41-59% of the time) | 2 |  |
|  |  |  |  | e) Infrequently (21-40% of the time) | 1 |  |
|  |  |  |  | f) Almost Never (1-20% of the time) | 0 |  |
|  |  |  |  | g) No multidisciplinary team rounds conducted | 0 |  |
|  |  |  | 3a. Are daily multidisciplinary patient care rounds led by a pediatric critical care physician or experienced pediatrician? | a) Yes, led by a pediatric critical care physician | 5 |  |
|  |  |  |  | b) Yes, led by an experienced pediatrician | 5 |  |
|  |  |  |  | c) No | 0 |  |
|  |  |  | 3b. Which team members are involved in the multidisciplinary patient care rounds: (Check all that apply) | a) ICU team | N/A |  |
|  |  |  |  | b) HO team |  |  |
|  |  |  |  | c) ICU Nurses |  |  |
|  |  |  |  | d) Surgical team (for surgical patients) |  |  |
|  |  |  |  | e) Pharmacy |  |  |
|  |  |  |  | f) Infectious disease team (when appropriate) |  |  |
|  |  |  |  | g) Respiratory therapist (when appropriate) |  |  |
|  |  |  |  | h) Nutritionist |  |  |
|  |  |  |  | i) Palliative care (when appropriate) |  |  |
|  |  |  |  | j) Parents |  |  |
|  |  |  |  | k) Other, please describe: |  |  |
|  |  |  | 4. How often are relevant test results, reports, and imaging studies available for review during daily rounds for hospitalized critically ill PHO patients? | a) Always (100% of the time) | 5 |  |
|  |  |  |  | b) Almost Always (80-99% of the time) | 4 |  |
|  |  |  |  | c) Frequently (60-79% of the time) | 3 |  |
|  |  |  |  | d) Sometimes/Moderate availability (41-59% of the time) | 2 |  |
|  |  |  |  | e) Infrequently/Limited availability (21-40% of the time) | 1 |  |
|  |  |  |  | f) Almost Never (1-20% of the time) | 0 |  |
|  |  |  |  | g) Never/Not available | 0 |  |
|  |  |  | 5. Is there a standardized system for signing out critically ill PHO patients between shifts, to give continuity of care every time responsibilities are transferred between physicians? | a) Yes | 5 |  |
|  |  |  |  | b) No | 0 |  |
|  |  | ONC | 1. How often are multidisciplinary team discussions regarding prognosis, plan of care, or goals of care, conducted (when indicated) prior to admitting a critically ill PHO patient to the PICU? | a) Always (100% of the time) | 5 |  |
|  |  |  |  | b) Almost Always (80-99% of the time) | 4 |  |
|  |  |  |  | c) Frequently (60-79% of the time) | 3 |  |
|  |  |  |  | d) Sometimes (41-59% of the time) | 2 |  |
|  |  |  |  | e) Infrequently (21-40% of the time) | 1 |  |
|  |  |  |  | f)  Almost Never (1-20% of the time) | 0 |  |
|  |  |  |  | g) Never/ No multidisciplinary team discussions | 0 |  |
|  |  |  | 2. How often are multidisciplinary team discussions regarding difficult critically ill PHO patient cases conducted (when indicated) to guide clinical or bioethical decisions? | a) Always (100% of the time) | 5 |  |
|  |  |  |  | b) Almost Always (80-99% of the time) | 4 |  |
|  |  |  |  | c) Frequently (60-79% of the time) | 3 |  |
|  |  |  |  | d) Sometimes (41-59% of the time) | 2 |  |

| Service Integration | Communication  and  Multidisciplinary  Care | ONC |  | e) Infrequently (21-40% of the time) | 1 |  |
| --- | --- | --- | --- | --- | --- | --- |
|  |  |  |  | f) Almost Never (1-20% of the time) | 0 |  |
|  |  |  |  | g) Never/ No multidisciplinary team discussions | 0 |  |
|  |  |  | 3. How often are palliative care services available when needed for the care of critically ill PHO patients? (In-person, by phone or telecommunication) | a) Always (100% of the time or 24 hrs. a day/7 day a week) | 5 |  |
|  |  |  |  | b) Almost Always (80-99% of the time or 24 hrs. Monday-Friday and some hours on the weekends) | 4 |  |
|  |  |  |  | c) Frequently (60-79% of the time or 24 hrs. Monday-Friday) | 3 |  |
|  |  |  |  | d) Sometimes/Moderate availability (41-59% of the time or only daytime 5-7 days a week) | 2 |  |
|  |  |  |  | e) Infrequently/Limited availability (21-40% of the time or only daytime 3-4 days a week) | 1 |  |
|  |  |  |  | f) Almost Never (1-20% of the time or only daytime 1-2 days a week) | 0 |  |
|  |  |  |  | g) Never/Not available | 0 |  |
|  |  |  | 4. How often are psychological/emotional support services available when needed for families of critically ill PHO patients? (In-person, by phone or telecommunication) | a) Always (100% of the time or 24 hrs. a day/7 day a week) | 5 |  |
|  |  |  |  | b) Almost Always (80-99% of the time or 24 hrs. Monday-Friday and some hours on the weekends) | 4 |  |
|  |  |  |  | c) Frequently (60-79% of the time or 24 hrs. Monday-Friday) | 3 |  |
|  |  |  |  | d) Sometimes/Moderate availability (41-59% of the time or only daytime 5-7 days a week) | 2 |  |
|  |  |  |  | e) Infrequently/Limited availability (21-40% of the time or only daytime 3-4 days a week) | 1 |  |
|  |  |  |  | f) Almost Never (1-20% of the time or only daytime 1-2 days a week) | 0 |  |
|  |  |  |  | g) Never/Not available | 0 |  |
|  | Parent involvement | PICU | 1. How often does your hospital allows unrestricted parent visitation for critically ill PHO patients at all times except when this is not in the best interest of the child and the family (e.g., during sterile procedures)? (Practices PRIOR to the COVID-19 pandemic) | a) Always (100% of the time) | 5 |  |
|  |  |  |  | b) Almost Always (80-99% of the time) | 4 |  |
|  |  |  |  | c) Frequently (60-79% of the time) | 3 |  |
|  |  |  |  | d) Sometimes (41-59% of the time) | 2 |  |
|  |  |  |  | e) Infrequently (21-40% of the time) | 1 |  |
|  |  |  |  | f) Almost Never (1-20% of the time) | 0 |  |
|  |  |  |  | g) No parental visitations allowed | 0 |  |
|  |  |  | 1a. Has parental visitations to critically ill PHO patients become more restricted (limited) during the COVID-19 pandemic? | a) Yes | 5 |  |
|  |  |  |  | b) No | 0 |  |
|  |  |  | 2. How often are multidisciplinary team discussions with parents of critically ill PHO patients held to discuss the clinical course of the patient? | a) Always (100% of the time) | 5 |  |
|  |  |  |  | b) Almost Always (80-99% of the time) | 4 |  |
|  |  |  |  | c) Frequently (60-79% of the time) | 3 |  |
|  |  |  |  | d) Sometimes (41-59% of the time) | 2 |  |
|  |  |  |  | e) Infrequently (21-40% of the time) | 1 |  |
|  |  |  |  | f) Almost Never (1-20% of the time) | 0 |  |
|  |  |  |  | g) No multidisciplinary discussions conducted | 0 |  |
|  |  |  | 3. Does your hospital have a system to measure parental satisfaction with the quality of care provided to their child in the PICU or area where critically ill PHO patients are treated? | a) Yes | 5 |  |
|  |  |  |  | b) No | 0 |  |
|  |  | ONC | 1. How often are multidisciplinary team discussions (care conferences) with parents of critically ill PHO patients held to discuss (when appropriate) futility of treatment, end of life or limitation of care? | a) Always (100% of the time) | 5 |  |
|  |  |  |  | b) Almost Always (80-99% of the time) | 4 |  |
|  |  |  |  | c) Frequently (60-79% of the time) | 3 |  |
|  |  |  |  | d) Sometimes (41-59% of the time) | 2 |  |
|  |  |  |  | e) Infrequently (21-40% of the time) | 1 |  |
|  |  |  |  | f) Almost Never (1-20% of the time) | 0 |  |
|  |  |  |  | g) Never/ No care conferences conducted | 0 |  |
|  |  |  | 2. How often are restrictions in the number of visitors and timing for visits removed for critically ill patients at the end-of life? (PRIOR to COVID-19 pandemic) | a) Always (100% of the time) | 5 |  |
|  |  |  |  | b) Almost Always (80-99% of the time) | 4 |  |
|  |  |  |  | c) Frequently (60-79% of the time) | 3 |  |
|  |  |  |  | d) Sometimes (41-59% of the time) | 2 |  |
|  |  |  |  | e) Infrequently (21-40% of the time) | 1 |  |
|  |  |  |  | f) Almost Never (1-20% of the time) | 0 |  |
|  |  |  |  | g) Never/ Not available | 0 |  |

| Service Integration | Quality Indicators | PICU | 1. Does your hospital have a system to track serious safety events and specific hospital acquired conditions in critically ill patients (including critically ill PHO patients)? (Check all that apply) | a) Adverse Drug Events | 5 |  |
| --- | --- | --- | --- | --- | --- | --- |
|  |  |  |  | b) Catheter-associated urinary tract infection (CAUTI) | 5 |  |
|  |  |  |  | c) Central line-associated bloodstream infection (CLABSI) | 5 |  |
|  |  |  |  | d) Ventilator-associated pneumonia (VAP) | 5 |  |
|  |  |  |  | e) Pressure ulcers and skin integrity | 5 |  |
|  |  |  |  | f) Surgical site infections | 5 |  |
|  |  |  |  | g) Injuries from falls | 5 |  |
|  |  |  |  | h) Peripheral intravenous infiltration and extravasations | 5 |  |
|  |  |  |  | i) Unplanned Extubations | 5 |  |
|  |  |  |  | j) Deep Venous Thrombosis (DVT) | 5 |  |
|  |  |  |  | k) No system in place to track these events | 0 |  |
|  |  |  | 2. Does your hospital have a system in place to evaluate the daily need to continue or remove devices in critically ill patients (including critically ill PHO patients)? | a) Yes | 5 |  |
|  |  |  |  | b) No | 0 |  |
|  |  |  | 3. Does you hospital have a protocol to prevent deep venous thrombosis in critically ill patients (including critically ill PHO patients)? | a) Yes | 5 |  |
|  |  |  |  | b) No | 0 |  |
|  |  |  | 4. Does you hospital have a protocol to prevent peptic ulcer in critically ill patients (including critically ill PHO patients)? | a) Yes | 5 |  |
|  |  |  |  | b) No | 0 |  |
|  |  |  | 5. Does you hospital have a protocol for daily oral care in critically ill patients (including critically ill PHO patients)? | a) Yes | 5 |  |
|  |  |  |  | b) No | 0 |  |
|  |  |  | 6. Does your hospital perform spontaneous breathing trials in mechanically ventilated patients (including critically ill PHO patients)? | a) Yes | 5 |  |
|  |  |  |  | b) No | 0 |  |
|  |  |  |  | c) No mechanical ventilation available | 0 |  |
|  |  |  | 7. Does your hospital have a system in place to evaluate the daily need to continue, discontinue or change current antimicrobial treatment in critically ill patients (including critically ill PHO patients)? | a) Yes | 5 |  |
|  |  |  |  | b) No | 0 |  |
|  |  |  | 8. Does your hospital have a standardized system to monitor and report abstinence and withdrawal scores in critically ill pediatric patients (including critically ill PHO patients)? | a) Yes | 5 |  |
|  |  |  |  | b) No | 0 |  |
|  |  |  | 9. Does your hospital have a standardized system to monitor and assess pain (e.g., Wong-Baker FACES scale) in critically ill pediatric patients (including critically ill PHO patients)? | a) Yes | 5 |  |
|  |  |  |  | b) No | 0 |  |
|  |  |  | 10. Does your hospital have a standardized system to monitor degree of sedation (e.g., RASS or SBS scores) and/or delirium (e.g., CAPD) for critically ill pediatric patients (including critically ill PHO patients)? | a) Yes | 5 |  |
|  |  |  |  | b) No | 0 |  |
|  |  |  | 11. Does your hospital have a system in place to evaluate the daily need to continue, discontinue or change current sedation medications in critically ill pediatric patients? | a) Yes | 5 |  |
|  |  |  |  | b) No | 0 |  |
|  |  |  | 12. Does your hospital have a surveillance system to track and report multi-drug resistant organisms (MDRO) in hospitalized PHO patients? | a) Yes | 5 |  |
|  |  |  |  | b) No | 0 |  |
| Supportive Services | General Supportive Services | PICU | 1. Does your hospital have a system or process to communicate/notify critical test results to the critical care team or the team caring for critically ill patients in a timely manner? (e.g., laboratory notifies bedside nurse) | a) Yes | 5 |  |
|  |  |  |  | b) No | 0 |  |
|  |  |  | 2. Does your hospital have an antibiotic stewardship to track antibiotic resistance organisms in critically ill pediatric patients (including critically ill PHO patients)? | a) Yes | 5 |  |
|  |  |  |  | b) No | 0 |  |
|  |  |  | 3. How often do equipment shortages and/or failures affect the management of critically ill PHO patients? | a) Always (100% of the time) | 0 |  |
|  |  |  |  | b) Almost Always (80-99% of the time) | 0 |  |
|  |  |  |  | c) Frequently (60-79% of the time) | 1 |  |
|  |  |  |  | d) Sometimes (41-59% of the time) | 2 |  |
|  |  |  |  | e) Infrequently (21-40% of the time) | 3 |  |
|  |  |  |  | f) Almost Never (1-20% of the time) | 4 |  |
|  |  |  |  | g) Never | 5 |  |

| Supportive Services | General Supportive Services | PICU | 4. How often does inadequate pediatric critical care nurse staffing affect the management of critically ill PHO patients)? | a) Always (100% of the time) | 0 |  |
| --- | --- | --- | --- | --- | --- | --- |
|  |  |  |  | b) Almost Always (80-99% of the time) | 0 |  |
|  |  |  |  | c) Frequently (60-79% of the time) | 1 |  |
|  |  |  |  | d) Sometimes (41-59% of the time) | 2 |  |
|  |  |  |  | e) Infrequently (21-40% of the time) | 3 |  |
|  |  |  |  | f) Almost Never (1-20% of the time) | 4 |  |
|  |  |  |  | g) Never | 5 |  |
|  |  |  | 5. How often do medication shortages affect the management of critically ill PHO patients)? | a) Always (100% of the time) | 0 |  |
|  |  |  |  | b) Almost Always (80-99% of the time) | 0 |  |
|  |  |  |  | c) Frequently (60-79% of the time) | 1 |  |
|  |  |  |  | d) Sometimes (41-59% of the time) | 2 |  |
|  |  |  |  | e) Infrequently (21-40% of the time) | 3 |  |
|  |  |  |  | f) Almost Never (1-20% of the time) | 4 |  |
|  |  |  |  | g) Never | 5 |  |
|  |  |  | 6. How often do blood product (packed red blood cells, platelets, fresh frozen plasma, etc.) shortages affect the management of critically ill PHO patients)? | a) Always (100% of the time) | 0 |  |
|  |  |  |  | b) Almost Always (80-99% of the time) | 0 |  |
|  |  |  |  | c) Frequently (60-79% of the time) | 1 |  |
|  |  |  |  | d) Sometimes (41-59% of the time) | 2 |  |
|  |  |  |  | e) Infrequently (21-40% of the time) | 3 |  |
|  |  |  |  | f) Almost Never (1-20% of the time) | 4 |  |
|  |  |  |  | g) Never | 5 |  |
|  |  |  | 7. Is there a standard system (operational plan) to monitor, prevent, and/or address medication shortages in the PICU (or area where critically ill PHO patients are managed)? | a) Yes | 5 |  |
|  |  |  |  | b) No | 0 |  |
|  | General Laboratory | PICU | 1. How often are pH and blood gas analysis studies available as needed for critically ill PHO patients)? | a) Always (24 hrs. a day/ 7 days a week or 100% of the time) | 5 |  |
|  |  |  |  | b) Almost Always (80-99% of the time) | 4 |  |
|  |  |  |  | c) Frequently (60-79% of the time) | 3 |  |
|  |  |  |  | d) Sometimes/Moderate availability (41-59% of the time) | 2 |  |
|  |  |  |  | e) Infrequently/Limited availability (21-40% of the time) | 1 |  |
|  |  |  |  | f) Almost Never (1-20% of the time) | 0 |  |
|  |  |  |  | g) Never/Not available | 0 |  |
|  |  |  | 2. How often are blood cell count (CBC + differential) studies available as needed for critically ill PHO patients? | a) Always (24 hrs. a day/ 7 days a week or 100% of the time) | 5 |  |
|  |  |  |  | b) Almost Always (80-99% of the time) | 4 |  |
|  |  |  |  | c) Frequently (60-79% of the time) | 3 |  |
|  |  |  |  | d) Sometimes/Moderate availability (41-59% of the time) | 2 |  |
|  |  |  |  | e) Infrequently/Limited availability (21-40% of the time) | 1 |  |
|  |  |  |  | f) Almost Never (1-20% of the time) | 0 |  |
|  |  |  |  | g) Never/Not available | 0 |  |
|  |  |  | 3. How often are Complete metabolic panel studies (electrolytes, glucose, kidney, and liver function) available as needed for critically ill PHO patients? | a) Always (24 hrs. a day/ 7 days a week or 100% of the time) | 5 |  |
|  |  |  |  | b) Almost Always (80-99% of the time) | 4 |  |
|  |  |  |  | c)  Frequently (60-79% of the time) | 3 |  |
|  |  |  |  | d) Sometimes/Moderate availability (41-59% of the time) | 2 |  |
|  |  |  |  | e) Infrequently/Limited availability (21-40% of the time) | 1 |  |
|  |  |  |  | f) Almost Never (1-20% of the time) | 0 |  |
|  |  |  |  | g) Never/Not available | 0 |  |
|  |  |  | 4. How often are coagulation studies (INR, PTT) available as needed for critically ill PHO patients? | a) Always (24 hrs. a day/ 7 days a week or 100% of the time) | 5 |  |
|  |  |  |  | b) Almost Always (80-99% of the time) | 4 |  |
|  |  |  |  | c) Frequently (60-79% of the time) | 3 |  |
|  |  |  |  | d) Sometimes/Moderate availability (41-59% of the time) | 2 |  |
|  |  |  |  | e) Infrequently/Limited availability (21-40% of the time) | 1 |  |
|  |  |  |  | f) Almost Never (1-20% of the time) | 0 |  |
|  |  |  |  | g) Never/Not available | 0 |  |

| Supportive Services | General Laboratory | PICU | 5. How often are LDH, Uric acid available as needed for critically ill PHO patients? | a) Always (24 hrs. a day/ 7 days a week or 100% of the time) | 5 |  |
| --- | --- | --- | --- | --- | --- | --- |
|  |  |  |  | b) Almost Always (80-99% of the time) | 4 |  |
|  |  |  |  | c) Frequently (60-79% of the time) | 3 |  |
|  |  |  |  | d) Sometimes/Moderate availability (41-59% of the time) | 2 |  |
|  |  |  |  | e) Infrequently/Limited availability (21-40% of the time) | 1 |  |
|  |  |  |  | f) Almost Never (1-20% of the time) | 0 |  |
|  |  |  |  | g) Never/Not available | 0 |  |
|  |  |  | 6. How often are urine analysis studies available as needed for critically ill PHO patients? | a) Always (24 hrs. a day/ 7 days a week or 100% of the time) | 5 |  |
|  |  |  |  | b) Almost Always (80-99% of the time) | 4 |  |
|  |  |  |  | c) Frequently (60-79% of the time) | 3 |  |
|  |  |  |  | d) Sometimes/Moderate availability (41-59% of the time) | 2 |  |
|  |  |  |  | e) Infrequently/Limited availability (21-40% of the time) | 1 |  |
|  |  |  |  | f) Almost Never (1-20% of the time) | 0 |  |
|  |  |  |  | g) Never/Not available | 0 |  |
|  |  |  | 7. In general, what is the turn-around time (reporting time) for STAT (emergency) laboratory test results? | a) < 1 hr. | 5 |  |
|  |  |  |  | b) 1-4 hrs. | 3 |  |
|  |  |  |  | c) 4-8 hrs. | 2 |  |
|  |  |  |  | d) 8-12 hrs. | 1 |  |
|  |  |  |  | e) > 12 hrs. | 0 |  |
|  |  |  | 8. How often are microbiology services for the identification of organisms in blood, urine or CSF available as needed for critically ill PHO patients? | a) Always (24 hrs. a day/ 7 days a week or 100% of the time) | 5 |  |
|  |  |  |  | b) Almost Always (80-99% of the time) | 4 |  |
|  |  |  |  | c) Frequently (60-79% of the time) | 3 |  |
|  |  |  |  | d) Sometimes/Moderate availability (41-59% of the time) | 2 |  |
|  |  |  |  | e) Infrequently/Limited availability (21-40% of the time) | 1 |  |
|  |  |  |  | f) Almost Never (1-20% of the time) | 0 |  |
|  |  |  |  | g) Never/Not available | 0 |  |
|  |  |  | 9. How often are antibiotic sensitivity analyses available as needed for critically ill PHO patients? | a) Always (24 hrs. a day/ 7 days a week or 100% of the time) | 5 |  |
|  |  |  |  | b) Almost Always (80-99% of the time) | 4 |  |
|  |  |  |  | c) Frequently (60-79% of the time) | 3 |  |
|  |  |  |  | d) Sometimes/Moderate availability (41-59% of the time) | 2 |  |
|  |  |  |  | e) Infrequently/Limited availability (21-40% of the time) | 1 |  |
|  |  |  |  | f) Almost Never (1-20% of the time) | 0 |  |
|  |  |  |  | g) Never/Not available | 0 |  |
|  |  |  | 10. How often are fungal cultures analyses available as needed for critically ill PHO patients? | a) Always (24 hrs. a day/ 7 days a week or 100% of the time) | 5 |  |
|  |  |  |  | b) Almost Always (80-99% of the time) | 4 |  |
|  |  |  |  | c) Frequently (60-79% of the time) | 3 |  |
|  |  |  |  | d) Sometimes/Moderate availability (41-59% of the time) | 2 |  |
|  |  |  |  | e) Infrequently/Limited availability (21-40% of the time) | 1 |  |
|  |  |  |  | f) Almost Never (1-20% of the time) | 0 |  |
|  |  |  |  | g) Never/Not available | 0 |  |
|  |  |  | 11. How often is monitoring of antimicrobial drug levels (e.g., vancomycin level) available as needed for critically ill PHO patients? | a) Always (24 hrs. a day/ 7 days a week or 100% of the time) | 5 |  |
|  |  |  |  | b) Almost Always (80-99% of the time) | 4 |  |
|  |  |  |  | c) Frequently (60-79% of the time) | 3 |  |
|  |  |  |  | d) Sometimes/Moderate availability (41-59% of the time) | 2 |  |
|  |  |  |  | e) Infrequently/Limited availability (21-40% of the time) | 1 |  |
|  |  |  |  | f) Almost Never (1-20% of the time) | 0 |  |
|  |  |  |  | g) Never/Not available | 0 |  |

| Supportive Services | General Laboratory | PICU | 12. How often is monitoring of antineoplastic drug levels (e.g., methotrexate levels) available as needed for critically ill PHO patients at your hospital? | a) Always (24 hrs. a day/ 7 days a week or 100% of the time) | 5 |  |
| --- | --- | --- | --- | --- | --- | --- |
|  |  |  |  | b) Almost Always (80-99% of the time) | 4 |  |
|  |  |  |  | c) Frequently (60-79% of the time) | 3 |  |
|  |  |  |  | d) Sometimes/Moderate availability (41-59% of the time) | 2 |  |
|  |  |  |  | e) Infrequently/Limited availability (21-40% of the time) | 1 |  |
|  |  |  |  | f) Almost Never (1-20% of the time) | 0 |  |
|  |  |  |  | g) Never/Not available | 0 |  |
|  |  |  | 13. Is there a system to perform drug-dosing adjustments in critically ill pediatric patients with kidney or liver disease? (e.g., computerized support alerts) | a) Yes | 5 |  |
|  |  |  |  | b) No | 0 |  |
|  | Imaging Services | PICU | 1. How often are portable x-rays available to perform STAT (emergency) imaging studies at the bedside for critically ill PHO patients? | a) Always (24 hrs. a day/ 7 days a week or 100% of the time) | 5 |  |
|  |  |  |  | b) Almost Always (80-99% of the time) | 4 |  |
|  |  |  |  | c) Frequently (60-79% of the time) | 3 |  |
|  |  |  |  | d) Sometimes/Moderate availability (41-59% of the time) | 2 |  |
|  |  |  |  | e) Infrequently/Limited availability (21-40% of the time) | 1 |  |
|  |  |  |  | f) Almost Never (1-20% of the time) | 0 |  |
|  |  |  |  | g) Never/Not available | 0 |  |
|  |  |  | 2. How often are portable ultrasound studies available to perform STAT (emergency) imaging studies at the bedside for critically ill PHO patients? | a) Always (24 hrs. a day/ 7 days a week or 100% of the time) | 5 |  |
|  |  |  |  | b) Almost Always (80-99% of the time) | 4 |  |
|  |  |  |  | c) Frequently (60-79% of the time) | 3 |  |
|  |  |  |  | d) Sometimes/Moderate availability (41-59% of the time) | 2 |  |
|  |  |  |  | e) Infrequently/Limited availability (21-40% of the time) | 1 |  |
|  |  |  |  | f) Almost Never (1-20% of the time) | 0 |  |
|  |  |  |  | g) Never/Not available | 0 |  |
|  |  |  | 3. How often are portable echocardiogram studies available to perform STAT (emergency) imaging studies at the bedside for critically ill PHO patients? | a) Always (24 hrs. a day/ 7 days a week or 100% of the time) | 5 |  |
|  |  |  |  | b) Almost Always (80-99% of the time) | 4 |  |
|  |  |  |  | c) Frequently (60-79% of the time) | 3 |  |
|  |  |  |  | d) Sometimes/Moderate availability (41-59% of the time) | 2 |  |
|  |  |  |  | e) Infrequently/Limited availability (21-40% of the time) | 1 |  |
|  |  |  |  | f) Almost Never (1-20% of the time) | 0 |  |
|  |  |  |  | g) Never/Not available | 0 |  |
|  |  |  | 4. How often is point-of-care ultrasound device available for use in critically ill PHO patients to assist with invasive procedures (e.g., central line placement) and augment physical examination findings (e.g., assessment for pulmonary effusion)? | a) Always (24 hrs. a day/ 7 days a week or 100% of the time) | 5 |  |
|  |  |  |  | b) Almost Always (80-99% of the time) | 4 |  |
|  |  |  |  | c) Frequently (60-79% of the time) | 3 |  |
|  |  |  |  | d) Sometimes/Moderate availability (41-59% of the time) | 2 |  |
|  |  |  |  | e) Infrequently/Limited availability (21-40% of the time) | 1 |  |
|  |  |  |  | f) Almost Never (1-20% of the time) | 0 |  |
|  |  |  |  | g) Never/Not available | 0 |  |
|  |  | ONC | 1. How often are not portable Plain XR (2 views) studies available for critically ill PHO patients? | a) Always (24 hrs. a day/ 7 days a week or 100% of the time) | 5 |  |
|  |  |  |  | b) Almost Always (80-99% of the time) | 4 |  |
|  |  |  |  | c) Frequently (60-79% of the time) | 3 |  |
|  |  |  |  | d) Sometimes/Moderate availability (41-59% of the time) | 2 |  |
|  |  |  |  | e) Infrequently/Limited availability (21-40% of the time) | 1 |  |
|  |  |  |  | f) Almost Never (1-20% of the time) | 0 |  |
|  |  |  |  | g) Never/Not available | 0 |  |
|  |  |  | 1a. In general, how long does it take for Plain XR to be done (from the time of ordering to the study actually being performed)? | a) < 24 hr. | 5 |  |
|  |  |  |  | b) 24-48 hrs. | 1 |  |
|  |  |  |  | c) > 48 hr. | 0 |  |

| Supportive Services | Imaging Services | ONC | 2. How often are Ultrasound studies available for critically ill PHO patient? | a) Always (24 hrs. a day/ 7 days a week or 100% of the time) | 5 |  |
| --- | --- | --- | --- | --- | --- | --- |
|  |  |  |  | b) Almost Always (80-99% of the time) | 4 |  |
|  |  |  |  | c) Frequently (60-79% of the time) | 3 |  |
|  |  |  |  | d) Sometimes/Moderate availability (41-59% of the time) | 2 |  |
|  |  |  |  | e) Infrequently/Limited availability (21-40% of the time) | 1 |  |
|  |  |  |  | f) Almost Never (1-20% of the time) | 0 |  |
|  |  |  |  | g) Never/Not available | 0 |  |
|  |  |  | 2a. In general, how long does it take for ultrasound studies to be done (from time of ordering to the study actually being performed)? | a) < 24 hr. | 5 |  |
|  |  |  |  | b) 24-4 8hrs. | 1 |  |
|  |  |  |  | c) > 48 hr. | 0 |  |
|  |  |  | 3. How often are Computed Tomography (CT) scans available for critically ill PHO patients)? | a) Always (24 hrs. a day/ 7 days a week or 100% of the time) | 5 |  |
|  |  |  |  | b) Almost Always (80-99% of the time) | 4 |  |
|  |  |  |  | c) Frequently (60-79% of the time) | 3 |  |
|  |  |  |  | d) Sometimes/Moderate availability (41-59% of the time) | 2 |  |
|  |  |  |  | e) Infrequently/Limited availability (21-40% of the time) | 1 |  |
|  |  |  |  | f) Almost Never (1-20% of the time) | 0 |  |
|  |  |  |  | g) Never/Not available | 0 |  |
|  |  |  | If the answer is G for question 3, skip this question, otherwise: 3a. In general, how long does it take for CT scans to be done (from time of ordering to the study actually being performed)? | a) < 24 hr. | 5 |  |
|  |  |  |  | b) 24-48 hrs. | 1 |  |
|  |  |  |  | c) > 48 hr. | 0 |  |
|  | Blood Bank/  Transfusion Services | PICU | 1. How often are Packed Red Blood Cells available as needed for the management of critically ill PHO patients? | a) Always (24 hrs. a day/ 7 days a week or 100% of the time) | 5 |  |
|  |  |  |  | b) Almost Always (80-99% of the time) | 4 |  |
|  |  |  |  | c) Frequently (60-79% of the time) | 3 |  |
|  |  |  |  | d) Sometimes/Moderate availability (41-59% of the time) | 2 |  |
|  |  |  |  | e) Infrequently/Limited availability (21-40% of the time) | 1 |  |
|  |  |  |  | f) Almost Never (1-20% of the time) | 0 |  |
|  |  |  |  | g) Never/Not available | 0 |  |
|  |  |  | 2. How often are Platelets available as needed for the management of critically ill PHO patients? | a) Always (24 hrs. a day/ 7 days a week or 100% of the time) | 5 |  |
|  |  |  |  | b) Almost Always (80-99% of the time) | 4 |  |
|  |  |  |  | c) Frequently (60-79% of the time) | 3 |  |
|  |  |  |  | d) Sometimes/Moderate availability (41-59% of the time) | 2 |  |
|  |  |  |  | e) Infrequently/Limited availability (21-40% of the time) | 1 |  |
|  |  |  |  | f) Almost Never (1-20% of the time) | 0 |  |
|  |  |  |  | g) Never/Not available | 0 |  |
|  |  |  | 3. How often is Fresh Frozen Plasma available as needed for the management of critically ill PHO patients? | a) Always (24 hrs. a day/ 7 days a week or 100% of the time) | 5 |  |
|  |  |  |  | b) Almost Always (80-99% of the time) | 4 |  |
|  |  |  |  | c) Frequently (60-79% of the time) | 3 |  |
|  |  |  |  | d) Sometimes/Moderate availability (41-59% of the time) | 2 |  |
|  |  |  |  | e) Infrequently/Limited availability (21-40% of the time) | 1 |  |
|  |  |  |  | f) Almost Never (1-20% of the time) | 0 |  |
|  |  |  |  | g) Never/Not available | 0 |  |
|  |  |  | 4. How often is Cryoprecipitate available as needed for the management of critically ill PHO patients? | a) Always (24 hrs. a day/ 7 days a week or 100% of the time) | 5 |  |
|  |  |  |  | b) Almost Always (80-99% of the time) | 4 |  |
|  |  |  |  | c) Frequently (60-79% of the time) | 3 |  |
|  |  |  |  | d) Sometimes/Moderate availability (41-59% of the time) | 2 |  |
|  |  |  |  | e) Infrequently/Limited availability (21-40% of the time) | 1 |  |
|  |  |  |  | f) Almost Never (1-20% of the time) | 0 |  |
|  |  |  |  | g) Never/Not available | 0 |  |

| Supportive Services | Blood Bank/  Transfusion Services | PICU | 5. How often is Leuco-depleted blood available as needed for the management of critically ill PHO patients? | a) Always (24 hrs. a day/ 7 days a week or 100% of the time) | 5 |  |
| --- | --- | --- | --- | --- | --- | --- |
|  |  |  |  | b) Almost Always (80-99% of the time) | 4 |  |
|  |  |  |  | c) Frequently (60-79% of the time) | 3 |  |
|  |  |  |  | d) Sometimes/Moderate availability (41-59% of the time) | 2 |  |
|  |  |  |  | e) Infrequently/Limited availability (21-40% of the time) | 1 |  |
|  |  |  |  | f) Almost Never (1-20% of the time) | 0 |  |
|  |  |  |  | g) Never/Not available | 0 |  |
|  |  |  | 6. In general, how long does it take to obtain STAT (emergency) blood products? | a) < 1 hr. | 5 |  |
|  |  |  |  | b) 1-4 hrs. | 3 |  |
|  |  |  |  | c) 5-8 hrs. | 2 |  |
|  |  |  |  | d) 9-12 hrs. | 1 |  |
|  |  |  |  | e) > 12 hrs. | 0 |  |
|  |  |  | 7. How often are blood type testing (e.g., ABO and RhD grouping) and cross matching pre-transfusion available as needed for the management of critically ill PHO patients? | a) Always (24 hrs. a day/ 7 days a week or 100% of the time) | 5 |  |
|  |  |  |  | b) Almost Always (80-99% of the time) | 4 |  |
|  |  |  |  | c) Frequently (60-79% of the time) | 3 |  |
|  |  |  |  | d) Sometimes/Moderate availability (41-59% of the time) | 2 |  |
|  |  |  |  | e) Infrequently/Limited availability (21-40% of the time) | 1 |  |
|  |  |  |  | f) Almost Never (1-20% of the time) | 0 |  |
|  |  |  |  | g) Never/Not available | 0 |  |
|  |  |  | 8. How often is blood product screening for infectious diseases (e.g., HIV) pre-transfusion available as needed for the management of critically ill PHO patients? | a) Always (24 hrs. a day/ 7 days a week or 100% of the time) | 5 |  |
|  |  |  |  | b) Almost Always (80-99% of the time) | 4 |  |
|  |  |  |  | c) Frequently (60-79% of the time) | 3 |  |
|  |  |  |  | d) Sometimes/Moderate availability (41-59% of the time) | 2 |  |
|  |  |  |  | e) Infrequently/Limited availability (21-40% of the time) | 1 |  |
|  |  |  |  | f) Almost Never (1-20% of the time) | 0 |  |
|  |  |  |  | g) Never/Not available | 0 |  |
|  | Operating Rooms/Anesthesia Services | PICU | 1. How often are acute surgery services (e.g., evaluation by a surgeon) available as needed for critically ill PHO patients with surgical emergencies? | a) Always (24 hrs. a day/ 7 days a week or 100% of the time) | 5 |  |
|  |  |  |  | b) Almost Always (80-99% of the time) | 4 |  |
|  |  |  |  | c) Frequently (60-79% of the time) | 3 |  |
|  |  |  |  | d) Sometimes/Moderate availability (41-59% of the time) | 2 |  |
|  |  |  |  | e) Infrequently/Limited availability (21-40% of the time) | 1 |  |
|  |  |  |  | f) Almost Never (1-20% of the time) | 0 |  |
|  |  |  |  | g) Never/Not available | 0 |  |
|  |  |  | 2. How often is an operating room available within 2hrs as needed for emergent surgeries in critically ill PHO patients? | a) Always (24 hrs. a day/ 7 days a week or 100% of the time) | 5 |  |
|  |  |  |  | b) Almost Always (80-99% of the time) | 4 |  |
|  |  |  |  | c) Frequently (60-79% of the time) | 3 |  |
|  |  |  |  | d) Sometimes/Moderate availability (41-59% of the time) | 2 |  |
|  |  |  |  | e) Infrequently/Limited availability (21-40% of the time) | 1 |  |
|  |  |  |  | f) Almost Never (1-20% of the time) | 0 |  |
|  |  |  |  | g) Never/No surgical services available at our hospital | 0 |  |
| Medication and Equipment | Medications | PICU | 1. How often are second or third-line antibiotics available for critically ill PHO patients with proven or suspected infection due to multi-drug resistant organisms? | a) Always (100% of the time) | 5 |  |
|  |  |  |  | b) Almost Always (80-99% of the time) | 4 |  |
|  |  |  |  | c) Frequently (60-79% of the time) | 3 |  |
|  |  |  |  | d) Sometimes/Moderate availability (41-59% of the time) | 2 |  |
|  |  |  |  | e) Infrequently/Limited availability (21-40% of the time) | 1 |  |
|  |  |  |  | f) Almost Never (1-20% of the time) | 0 |  |
|  |  |  |  | g) Never/Not available | 0 |  |

| Medication and Equipment | Medications | PICU | 2. How often are sedatives and analgesics (e.g., Fentanyl, Lorazepam) available when needed for critically ill PHO patients? | a) Always (100% of the time) | 5 |  |
| --- | --- | --- | --- | --- | --- | --- |
|  |  |  |  | b) Almost Always (80-99% of the time) | 4 |  |
|  |  |  |  | c) Frequently (60-79% of the time) | 3 |  |
|  |  |  |  | d) Sometimes/Moderate availability (41-59% of the time) | 2 |  |
|  |  |  |  | e) Infrequently/Limited availability (21-40% of the time) | 1 |  |
|  |  |  |  | f) Almost Never (1-20% of the time) | 0 |  |
|  |  |  |  | g) Never/Not available | 0 |  |
|  |  |  | 3. How often are anticonvulsants (e.g., Diazepam, Valproic Acid) available when needed for management for critically ill PHO patients)? | a) Always (100% of the time) | 5 |  |
|  |  |  |  | b) Almost Always (80-99% of the time) | 4 |  |
|  |  |  |  | c) Frequently (60-79% of the time) | 3 |  |
|  |  |  |  | d) Sometimes/Moderate availability (41-59% of the time) | 2 |  |
|  |  |  |  | e) Infrequently/Limited availability (21-40% of the time) | 1 |  |
|  |  |  |  | f) Almost Never (1-20% of the time) | 0 |  |
|  |  |  |  | g) Never/Not available | 0 |  |
|  |  |  | 4. How often are inotropes and vasoactive infusions (e.g., Dopamine, Epinephrine, Norepinephrine) available when needed for the management of critically ill PHO patients? | a) Always (100% of the time) | 5 |  |
|  |  |  |  | b) Almost Always (80-99% of the time) | 4 |  |
|  |  |  |  | c) Frequently (60-79% of the time) | 3 |  |
|  |  |  |  | d) Sometimes/Moderate availability (41-59% of the time) | 2 |  |
|  |  |  |  | e) Infrequently/Limited availability (21-40% of the time) | 1 |  |
|  |  |  |  | f) Almost Never (1-20% of the time) | 0 |  |
|  |  |  |  | g) Never/Not available | 0 |  |
|  |  |  | 5. How often are antiarrhythmic drugs (e.g., Adenosine, Amiodarone) available when needed for critically ill PHO patients? | a) Always (100% of the time) | 5 |  |
|  |  |  |  | b) Almost Always (80-99% of the time) | 4 |  |
|  |  |  |  | c) Frequently (60-79% of the time) | 3 |  |
|  |  |  |  | d) Sometimes/Moderate availability (41-59% of the time) | 2 |  |
|  |  |  |  | e) Infrequently/Limited availability (21-40% of the time) | 1 |  |
|  |  |  |  | f) Almost Never (1-20% of the time) | 0 |  |
|  |  |  |  | g) Never/Not available | 0 |  |
|  |  |  | 6. How often are medications to treat increased intracranial pressure (e.g., Mannitol, 3% hypertonic saline) available when needed for critically ill PHO patients? | a) Always (100% of the time) | 5 |  |
|  |  |  |  | b) Almost Always (80-99% of the time) | 4 |  |
|  |  |  |  | c) Frequently (60-79% of the time) | 3 |  |
|  |  |  |  | d) Sometimes/Moderate availability (41-59% of the time) | 2 |  |
|  |  |  |  | e) Infrequently/Limited availability (21-40% of the time) | 1 |  |
|  |  |  |  | f) Almost Never (1-20% of the time) | 0 |  |
|  |  |  |  | g) Never/Not available | 0 |  |
|  |  | ONC | 1. How often are first line antibiotics available for critically ill PHO patients presenting with fever and neutropenia? | a) Always (100% of the time) | 5 |  |
|  |  |  |  | b) Almost Always (80-99% of the time) | 4 |  |
|  |  |  |  | c) Frequently (60-79% of the time) | 3 |  |
|  |  |  |  | d) Sometimes/Moderate availability (41-59% of the time) | 2 |  |
|  |  |  |  | e) Infrequently/Limited availability (21-40% of the time) | 1 |  |
|  |  |  |  | f) Almost Never (1-20% of the time) | 0 |  |
|  |  |  |  | g) Never/Not available | 0 |  |
|  |  |  | 2. How often are antiemetics (e.g., Ondansetron) available when needed for management of critically ill PHO patients? | a) Always (100% of the time) | 5 |  |
|  |  |  |  | b) Almost Always (80-99% of the time) | 4 |  |
|  |  |  |  | c) Frequently (60-79% of the time) | 3 |  |
|  |  |  |  | d) Sometimes/Moderate availability (41-59% of the time) | 2 |  |
|  |  |  |  | e) Infrequently/Limited availability (21-40% of the time) | 1 |  |
|  |  |  |  | f) Almost Never (1-20% of the time) | 0 |  |
|  |  |  |  | g) Never/Not available | 0 |  |

| Medication and Equipment | Medications | ONC | 3. How often are IV isotonic fluids (e.g., Normal Saline, Ringer’s Lactate solution or Hartmann's solution) available when needed for management of critically ill PHO patients? | a) Always (100% of the time) | 5 |  |
| --- | --- | --- | --- | --- | --- | --- |
|  |  |  |  | b) Almost Always (80-99% of the time) | 4 |  |
|  |  |  |  | c) Frequently (60-79% of the time) | 3 |  |
|  |  |  |  | d) Sometimes/Moderate availability (41-59% of the time) | 2 |  |
|  |  |  |  | e) Infrequently/Limited availability (21-40% of the time) | 1 |  |
|  |  |  |  | f) Almost Never (1-20% of the time) | 0 |  |
|  |  |  |  | g) Never/Not available | 0 |  |
|  |  |  | 4. How often are diuretics (e.g., Furosemide) available when needed for management of critically ill PHO patients? | a) Always (100% of the time) | 5 |  |
|  |  |  |  | b) Almost Always (80-99% of the time) | 4 |  |
|  |  |  |  | c) Frequently (60-79% of the time) | 3 |  |
|  |  |  |  | d) Sometimes/Moderate availability (41-59% of the time) | 2 |  |
|  |  |  |  | e) Infrequently/Limited availability (21-40% of the time) | 1 |  |
|  |  |  |  | f) Almost Never (1-20% of the time) | 0 |  |
|  |  |  |  | g) Never/Not available | 0 |  |
|  |  |  | 5. How often is parental nutrition (PPN or TPN) available when needed for critically ill PHO patients? | a) Always (100% of the time) | 5 |  |
|  |  |  |  | b) Almost Always (80-99% of the time) | 4 |  |
|  |  |  |  | c) Frequently (60-79% of the time) | 3 |  |
|  |  |  |  | d) Sometimes/Moderate availability (41-59% of the time) | 2 |  |
|  |  |  |  | e) Infrequently/Limited availability (21-40% of the time) | 1 |  |
|  |  |  |  | f) Almost Never (1-20% of the time) | 0 |  |
|  |  |  |  | g) Never/Not available | 0 |  |
|  | Equipment and Supplies | PICU | 1. How often are vital sign monitoring equipment with alarms and continuous monitoring capabilities available at each bedside of critically ill PHO patients at your hospital? | a) Always (100% of the time) | 5 |  |
|  |  |  |  | b) Almost Always (80-99% of the time) | 4 |  |
|  |  |  |  | c) Frequently (60-79% of the time) | 3 |  |
|  |  |  |  | d) Sometimes/Moderate availability (41-59% of the time) | 2 |  |
|  |  |  |  | e) Infrequently/Limited availability (21-40% of the time) | 1 |  |
|  |  |  |  | f) Almost Never (1-20% of the time) | 0 |  |
|  |  |  |  | g) Never/Not available | 0 |  |
|  |  |  | 1a. If monitoring equipment available, are the clinical alarms: (Check all that apply) | a) Able to be customized for the needs of the individual patient | N/A |  |
|  |  |  |  | b) Alarms are audible, visually displayed and distinguishable over other noises |  |  |
|  |  |  |  | c) Presence of a system to report alarm failures/defective equipment |  |  |
|  |  |  |  | d) None of the above |  |  |
|  |  |  | 1b. If monitoring equipment available, is the healthcare personnel trained in the use and limitations of the equipment? | a) Yes | 5 |  |
|  |  |  |  | b) No | 0 |  |
|  |  |  |  | c) Do not know | 0 |  |
|  |  |  | 2. How often are Non-invasive blood pressure cuffs (infant, pediatric, adult size) for vital sign monitoring available as needed for management of critically ill PHO patients? | a) Always (100% of the time) | 5 |  |
|  |  |  |  | b) Almost Always (80-99% of the time) | 4 |  |
|  |  |  |  | c) Frequently (60-79% of the time) | 3 |  |
|  |  |  |  | d) Sometimes/Moderate availability (41-59% of the time) | 2 |  |
|  |  |  |  | e) Infrequently/Limited availability (21-40% of the time) | 1 |  |
|  |  |  |  | f) Almost Never (1-20% of the time) | 0 |  |
|  |  |  |  | g) Never/Not available | 0 |  |
|  |  |  | 3. How often are Pulse Oximetry probes (infant, pediatric, adult size) for vital sign monitoring available as needed for management of critically ill PHO patients? | a) Always (100% of the time) | 5 |  |
|  |  |  |  | b) Almost Always (80-99% of the time) | 4 |  |
|  |  |  |  | c) Frequently (60-79% of the time) | 3 |  |
|  |  |  |  | d) Sometimes/Moderate availability (41-59% of the time) | 2 |  |
|  |  |  |  | e) Infrequently/Limited availability (21-40% of the time) | 1 |  |
|  |  |  |  | f) Almost Never (1-20% of the time) | 0 |  |
|  |  |  |  | g) Never/Not available | 0 |  |

| Medication and Equipment | Equipment and Supplies | PICU | 4. How often are EKG leads for vital sign monitoring available as needed for management of critically ill PHO patients? | a) Always (100% of the time) | 5 |  |
| --- | --- | --- | --- | --- | --- | --- |
|  |  |  |  | b) Almost Always (80-99% of the time) | 4 |  |
|  |  |  |  | c) Frequently (60-79% of the time) | 3 |  |
|  |  |  |  | d) Sometimes/Moderate availability (41-59% of the time) | 2 |  |
|  |  |  |  | e) Infrequently/Limited availability (21-40% of the time) | 1 |  |
|  |  |  |  | f) Almost Never (1-20% of the time) | 0 |  |
|  |  |  |  | g) Never/Not available | 0 |  |
|  |  |  | 5. How often are Capnography (end tidal CO2) monitors available as needed for management of critically ill PHO patients? | a) Always (100% of the time) | 5 |  |
|  |  |  |  | b) Almost Always (80-99% of the time) | 4 |  |
|  |  |  |  | c) Frequently (60-79% of the time) | 3 |  |
|  |  |  |  | d) Sometimes/Moderate availability (41-59% of the time) | 2 |  |
|  |  |  |  | e) Infrequently/Limited availability (21-40% of the time) | 1 |  |
|  |  |  |  | f) Almost Never (1-20% of the time) | 0 |  |
|  |  |  |  | g) Never/Not available | 0 |  |
|  |  |  | 6. How often are thermometers available as needed for management of critically ill PHO patients? | a) Always (100% of the time) | 5 |  |
|  |  |  |  | b) Almost Always (80-99% of the time) | 4 |  |
|  |  |  |  | c) Frequently (60-79% of the time) | 3 |  |
|  |  |  |  | d) Sometimes/Moderate availability (41-59% of the time) | 2 |  |
|  |  |  |  | e) Infrequently/Limited availability (21-40% of the time) | 1 |  |
|  |  |  |  | f) Almost Never (1-20% of the time) | 0 |  |
|  |  |  |  | g) Never/Not available | 0 |  |
|  |  |  | 7. How often are critical supplies (crash carts, refrigerator and cabinets for medications, respiratory equipment) readily available within or immediately adjacent to the PICU (or area where critically ill PHO patients are managed) for rapid retrieval in emergencies? | a) Always (100% of the time) | 5 |  |
|  |  |  |  | b) Almost Always (80-99% of the time) | 4 |  |
|  |  |  |  | c) Frequently (60-79% of the time) | 3 |  |
|  |  |  |  | d) Sometimes/Moderate availability (41-59% of the time) | 2 |  |
|  |  |  |  | e) Infrequently/Limited availability (21-40% of the time) | 1 |  |
|  |  |  |  | f) Almost Never (1-20% of the time) | 0 |  |
|  |  |  |  | g) Never/Not available | 0 |  |
|  |  |  | 8. How often are pediatric-sized oro- and nasopharyngeal airways equipment available, as needed for management of critically ill PHO patients? | a) Always (100% of the time) | 5 |  |
|  |  |  |  | b) Almost Always (80-99% of the time) | 4 |  |
|  |  |  |  | c) Frequently (60-79% of the time) | 3 |  |
|  |  |  |  | d) Sometimes/Moderate availability (41-59% of the time) | 2 |  |
|  |  |  |  | e) Infrequently/Limited availability (21-40% of the time) | 1 |  |
|  |  |  |  | f) Almost Never (1-20% of the time) | 0 |  |
|  |  |  |  | g) Never/Not available | 0 |  |
|  |  |  | 9. How often are pediatric-sized anesthesia (free flow) or self-inflating ventilation (ambu) bags and masks available, as needed for management of critically ill PHO patients? | a) Always (100% of the time) | 5 |  |
|  |  |  |  | b) Almost Always (80-99% of the time) | 4 |  |
|  |  |  |  | c) Frequently (60-79% of the time) | 3 |  |
|  |  |  |  | d) Sometimes/Moderate availability (41-59% of the time) | 2 |  |
|  |  |  |  | e) Infrequently/Limited availability (21-40% of the time) | 1 |  |
|  |  |  |  | f) Almost Never (1-20% of the time) | 0 |  |
|  |  |  |  | g) Never/Not available | 0 |  |
|  |  |  | 10. How often are pediatric-sized High flow nasal prongs available, as needed for management of critically ill PHO patients? | a) Always (100% of the time) | 5 |  |
|  |  |  |  | b) Almost Always (80-99% of the time) | 4 |  |
|  |  |  |  | c) Frequently (60-79% of the time) | 3 |  |
|  |  |  |  | d) Sometimes/Moderate availability (41-59% of the time) | 2 |  |
|  |  |  |  | e) Infrequently/Limited availability (21-40% of the time) | 1 |  |
|  |  |  |  | f) Almost Never (1-20% of the time) | 0 |  |
|  |  |  |  | g) Never/Not available | 0 |  |

| Medication and Equipment | Equipment and Supplies | PICU | 11. How often are pediatric-sized interfaces masks for Non-invasive ventilation (CPAP and BiPAP) available, as needed for management of critically ill PHO patients? | a) Always (100% of the time) | 5 |  |
| --- | --- | --- | --- | --- | --- | --- |
|  |  |  |  | b) Almost Always (80-99% of the time) | 4 |  |
|  |  |  |  | c) Frequently (60-79% of the time) | 3 |  |
|  |  |  |  | d) Sometimes/Moderate availability (41-59% of the time) | 2 |  |
|  |  |  |  | e) Infrequently/Limited availability (21-40% of the time) | 1 |  |
|  |  |  |  | f) Almost Never (1-20% of the time) | 0 |  |
|  |  |  |  | g) Never/Not available | 0 |  |
|  |  |  | 12. How often are pediatric-sized endotracheal tubes (ETT) and ETT suction catheters available, as needed for management of critically ill PHO patients? | a) Always (100% of the time) | 5 |  |
|  |  |  |  | b) Almost Always (80-99% of the time) | 4 |  |
|  |  |  |  | c) Frequently (60-79% of the time) | 3 |  |
|  |  |  |  | d) Sometimes/Moderate availability (41-59% of the time) | 2 |  |
|  |  |  |  | e) Infrequently/Limited availability (21-40% of the time) | 1 |  |
|  |  |  |  | f) Almost Never (1-20% of the time) | 0 |  |
|  |  |  |  | g) Never/Not available | 0 |  |
|  |  |  | 13. How often are pediatric-sized Laryngoscope blades available, as needed for management of critically ill PHO patients? | a) Always (100% of the time) | 5 |  |
|  |  |  |  | b) Almost Always (80-99% of the time) | 4 |  |
|  |  |  |  | c) Frequently (60-79% of the time) | 3 |  |
|  |  |  |  | d) Sometimes/Moderate availability (41-59% of the time) | 2 |  |
|  |  |  |  | e) Infrequently/Limited availability (21-40% of the time) | 1 |  |
|  |  |  |  | f) Almost Never (1-20% of the time) | 0 |  |
|  |  |  |  | g) Never/Not available | 0 |  |
|  |  |  | 14. How often are pediatric-sized ventilator circuits available, as needed for management of critically ill PHO patients? | a) Always (100% of the time) | 5 |  |
|  |  |  |  | b) Almost Always (80-99% of the time) | 4 |  |
|  |  |  |  | c) Frequently (60-79% of the time) | 3 |  |
|  |  |  |  | d) Sometimes/Moderate availability (41-59% of the time) | 2 |  |
|  |  |  |  | e) Infrequently/Limited availability (21-40% of the time) | 1 |  |
|  |  |  |  | f) Almost Never (1-20% of the time) | 0 |  |
|  |  |  |  | g) Never/Not available | 0 |  |
|  |  |  | 15. How often are pediatric-sized Laryngeal mask airways (LMAs) available, as needed for management of critically ill PHO patients? | a) Always (100% of the time) | 5 |  |
|  |  |  |  | b) Almost Always (80-99% of the time) | 4 |  |
|  |  |  |  | c) Frequently (60-79% of the time) | 3 |  |
|  |  |  |  | d) Sometimes/Moderate availability (41-59% of the time) | 2 |  |
|  |  |  |  | e) Infrequently/Limited availability (21-40% of the time) | 1 |  |
|  |  |  |  | f) Almost Never (1-20% of the time) | 0 |  |
|  |  |  |  | g) Never/Not available | 0 |  |
|  |  |  | 16. How often are intracranial pressuring (ICP) monitors available as needed for management of critically ill PHO? | a) Always (100% of the time) | 5 |  |
|  |  |  |  | b) Almost Always (80-99% of the time) | 4 |  |
|  |  |  |  | c) Frequently (60-79% of the time) | 3 |  |
|  |  |  |  | d) Sometimes/Moderate availability (41-59% of the time) | 2 |  |
|  |  |  |  | e) Infrequently/Limited availability (21-40% of the time) | 1 |  |
|  |  |  |  | f) Almost Never (1-20% of the time) | 0 |  |
|  |  |  |  | g) Never/Not available | 0 |  |
|  |  |  | 17. How often is renal replacement therapy (hemodialysis and/or peritoneal dialysis) available, as needed for management of critically ill PHO patients? | a) Always (100% of the time) | 5 |  |
|  |  |  |  | b) Almost Always (80-99% of the time) | 4 |  |
|  |  |  |  | c) Frequently (60-79% of the time) | 3 |  |
|  |  |  |  | d) Sometimes/Moderate availability (41-59% of the time) | 2 |  |
|  |  |  |  | e) Infrequently/Limited availability (21-40% of the time) | 1 |  |
|  |  |  |  | f) Almost Never (1-20% of the time) | 0 |  |
|  |  |  |  | g) Never/Not available | 0 |  |

| Medication and Equipment | Equipment and Supplies | PICU | 18. How often are pediatric-sized Intra-osseous (IO) needles available as needed for management of critically ill PHO patients? | a) Always (100% of the time) | 5 |  |
| --- | --- | --- | --- | --- | --- | --- |
|  |  |  |  | b) Almost Always (80-99% of the time) | 4 |  |
|  |  |  |  | c) Frequently (60-79% of the time) | 3 |  |
|  |  |  |  | d) Sometimes/Moderate availability (41-59% of the time) | 2 |  |
|  |  |  |  | e) Infrequently/Limited availability (21-40% of the time) | 1 |  |
|  |  |  |  | f) Almost Never (1-20% of the time) | 0 |  |
|  |  |  |  | g) Never/Not available | 0 |  |
|  |  |  | 19. How often are pediatric-sized arterial line catheters available as needed for management of critically ill PHO patients? | a) Always (100% of the time) | 5 |  |
|  |  |  |  | b) Almost Always (80-99% of the time) | 4 |  |
|  |  |  |  | c) Frequently (60-79% of the time) | 3 |  |
|  |  |  |  | d) Sometimes/Moderate availability (41-59% of the time) | 2 |  |
|  |  |  |  | e) Infrequently/Limited availability (21-40% of the time) | 1 |  |
|  |  |  |  | f) Almost Never (1-20% of the time) | 0 |  |
|  |  |  |  | g) Never/Not available | 0 |  |
|  |  |  | 20. How often are pediatric-sized Central line catheters available as needed for management of critically ill PHO patients? | a) Always (100% of the time) | 5 |  |
|  |  |  |  | b) Almost Always (80-99% of the time) | 4 |  |
|  |  |  |  | c) Frequently (60-79% of the time) | 3 |  |
|  |  |  |  | d) Sometimes/Moderate availability (41-59% of the time) | 2 |  |
|  |  |  |  | e) Infrequently/Limited availability (21-40% of the time) | 1 |  |
|  |  |  |  | f) Almost Never (1-20% of the time) | 0 |  |
|  |  |  |  | g) Never/Not available | 0 |  |
|  |  |  | 21. How often are pediatric-sized Chest tubes available as needed for management of critically ill PHO patients? | a) Always (100% of the time) | 5 |  |
|  |  |  |  | b) Almost Always (80-99% of the time) | 4 |  |
|  |  |  |  | c) Frequently (60-79% of the time) | 3 |  |
|  |  |  |  | d) Sometimes/Moderate availability (41-59% of the time) | 2 |  |
|  |  |  |  | e) Infrequently/Limited availability (21-40% of the time) | 1 |  |
|  |  |  |  | f) Almost Never (1-20% of the time) | 0 |  |
|  |  |  |  | g) Never/Not available | 0 |  |
|  |  |  | 22. How often are pediatric-sized Urinary catheters available as needed for management of critically ill PHO patients? | a) Always (100% of the time) | 5 |  |
|  |  |  |  | b) Almost Always (80-99% of the time) | 4 |  |
|  |  |  |  | c) Frequently (60-79% of the time) | 3 |  |
|  |  |  |  | d) Sometimes/Moderate availability (41-59% of the time) | 2 |  |
|  |  |  |  | e) Infrequently/Limited availability (21-40% of the time) | 1 |  |
|  |  |  |  | f) Almost Never (1-20% of the time) | 0 |  |
|  |  |  |  | g) Never/Not available | 0 |  |
|  |  |  | 23. Is there an equipment maintenance and servicing department at your hospital? | a) Yes | 3 |  |
|  |  |  |  | b) No | 0 |  |
|  |  |  |  | c) Do not know | 0 |  |
|  |  | ONC | 1. How often are pediatric-sized oxygen and aerosol/nebulization therapy masks (venturi mask, non-rebreather masks, nasal cannulas) available, as needed for management of critically ill PHO patients? | a) Always (100% of the time) | 5 |  |
|  |  |  |  | b) Almost Always (80-99% of the time) | 4 |  |
|  |  |  |  | c) Frequently (60-79% of the time) | 3 |  |
|  |  |  |  | d) Sometimes/Moderate availability (41-59% of the time) | 2 |  |
|  |  |  |  | e) Infrequently/Limited availability (21-40% of the time) | 1 |  |
|  |  |  |  | f) Almost Never (1-20% of the time) | 0 |  |
|  |  |  |  | g) Never/Not available | 0 |  |
|  |  |  | 2. How often are pediatric-sized peripheral intravenous (PIV) catheters available as needed for management of critically ill PHO patients? | a) Always (100% of the time) | 5 |  |
|  |  |  |  | b) Almost Always (80-99% of the time) | 4 |  |
|  |  |  |  | c) Frequently (60-79% of the time) | 3 |  |

| Medication and Equipment | Equipment and Supplies | ONC |  | d) Sometimes/Moderate availability (41-59% of the time) | 2 |  |
| --- | --- | --- | --- | --- | --- | --- |
|  |  |  |  | e) Infrequently/Limited availability (21-40% of the time) | 1 |  |
|  |  |  |  | f) Almost Never (1-20% of the time) | 0 |  |
|  |  |  |  | g) Never/Not available | 0 |  |
|  |  |  | 3. How often are pediatric-sized nasogastric tubes available as needed for management of critically ill PHO patients? | a) Always (100% of the time) | 5 |  |
|  |  |  |  | b) Almost Always (80-99% of the time) | 4 |  |
|  |  |  |  | c) Frequently (60-79% of the time) | 3 |  |
|  |  |  |  | d) Sometimes/Moderate availability (41-59% of the time) | 2 |  |
|  |  |  |  | e) Infrequently/Limited availability (21-40% of the time) | 1 |  |
|  |  |  |  | f) Almost Never (1-20% of the time) | 0 |  |
|  |  |  |  | g) Never/Not available | 0 |  |
| Outcomes | Diagnosis and Outcomes | PICU | 1. How often are multidisciplinary meetings conducted to review resource utilization (e.g., mechanical ventilation days, urinary catheter days, iNO days, etc.) at your hospital? | a) Monthly or more frequently | 5 |  |
|  |  |  |  | b) Quarterly (Every 3 months) | 3 |  |
|  |  |  |  | c) Twice a year (Every 6 months) | 1 |  |
|  |  |  |  | d) Yearly | 0 |  |
|  |  |  |  | e) We do not have such meetings | 0 |  |
|  |  |  |  | f) Do not know | 0 |  |
|  |  |  | 2. Does your hospital have a patient data registry that includes hospitalized PHO/BMT patients? | a) Yes, registry includes data for PHO/BMT patients | 5 |  |
|  |  |  |  | b) Yes, but the registry does not include data for PHO/BMT patients | 3 |  |
|  |  |  |  | c) We do not have a registry | 0 |  |
|  |  |  |  | d) Do not know | 0 |  |
|  |  |  | 3. What is the total number of pediatric patient admitted to the PICU/IMCU/HDU (or area where critically ill patients are managed) during the past 12 months? (In the comments provide data source or if data is not available) | a) _________ (number) | N/A |  |
|  |  |  |  | Comments: (e.g., hospital registry, data manager, no data available): |  |  |
|  |  |  | 4. What is the total number of pediatric patient who have died in the PICU/IMCU/HDU (or area where critically ill patients are managed) during the past 12 months? (In the comments provide data source or if data is not available) | a) _________ (number) | N/A |  |
|  |  |  |  | Comments: (e.g., hospital registry, data manager, no data available): |  |  |
|  |  |  | 5. What is the total number of PHO/BMT patients admitted to the PICU/IMCU/HDU (or area where critically ill patients are managed) in the past 12 months? (In the comments provide data source or if data is not available) | a) _________ (number) | N/A |  |
|  |  |  |  | Comments: (e.g., hospital registry, data manager, no data available) |  |  |
|  |  |  | 6. What is the total number of PHO/BMT patients who have died in the PICU/IMCU/HDU (or area where critically ill patients are managed) during the past 12 months? (In the comments provide data source or if data is not available) | a) _________ (number) | N/A |  |
|  |  |  |  | Comments: (e.g., hospital registry, data manager, no data available) |  |  |
|  |  |  | 7. What is the total number of PHO/BMT patients who have died of sepsis in the PICU/IMCU/HDU (or area where critically ill patients are managed) during the past 12 months? (In the comments provide data source or if data is not available) | a) _________ (number) | N/A |  |
|  |  |  |  | Comments: (e.g., hospital registry, data manager, no data available) |  |  |
|  |  |  | 8. What is the total number of PHO/BMT patients who have died of respiratory failure in the PICU/IMCU/HDU (or areas where critically ill patients are managed) during the past 12 months? (In the comments provide data source or if data is not available) | a) _________ (number) | N/A |  |
|  |  |  |  | Comments: (e.g., hospital registry, data manager, no data available) |  |  |
|  |  | ONC | 1. Does your hospital collaborate with other regional or international centers to compare and benchmark outcomes for critically ill PHO patients? | a) Yes | 5 |  |
|  |  |  |  | b) No | 0 |  |
|  |  |  |  | c) Do not know | 0 |  |
|  |  |  | 2. How often are multidisciplinary meetings conducted to review morbidity and mortality cases for critically ill PHO patients at your hospital? | a) Monthly or more frequently | 5 |  |
|  |  |  |  | b) Quarterly (Every 3 months) | 3 |  |
|  |  |  |  | c) Twice a year (Every 6 months) | 1 |  |
|  |  |  |  | d) Yearly | 0 |  |
|  |  |  |  | e) We do not have such meetings | 0 |  |
|  |  |  |  | f) Do not know | 0 |  |

| Outcomes | Diagnosis and Outcomes | ONC | 3. How often are multidisciplinary quality and patient safety meetings conducted to review clinical incidents, near misses, adverse drug events and/or healthcare acquired conditions for critically ill PHO patients at your hospital? | a) Monthly or more frequently | 5 |  |
| --- | --- | --- | --- | --- | --- | --- |
|  |  |  |  | b) Quarterly (Every 3 months) | 3 |  |
|  |  |  |  | c) Twice a year (Every 6 months) | 1 |  |
|  |  |  |  | d Yearly | 0 |  |
|  |  |  |  | e) We do not have such meetings | 0 |  |
|  |  |  |  | f) Do not know | 0 |  |
|  |  |  | 4. What is the average number of pediatric hospital admissions per year (in the last 12 months) at your hospital? (In the comments provide data source or if data is not available) | a) _________ (number) | N/A |  |
|  |  |  |  | Comments: (e.g., hospital registry, data manager, no data available): |  |  |
|  |  |  | 5. What is the average number of PHO admissions per year (in the last 12 months) at your hospital? (In the comments provide data source or if data is not available) (In the comments provide data source or if data is not available) | a) _________ (number) | N/A |  |
|  |  |  |  | Comments: (e.g., hospital registry, data manager, no data available): |  |  |
|  |  |  | 6. What is the total number of newly diagnosed pediatric cancer patients treated at your hospital in the past 12 months? (In the comments provide data source or if data is not available) | a) _________ (number) | N/A |  |
|  |  |  |  | Comments: (e.g., hospital registry, data manager, no data available): |  |  |
|  |  |  | 7. What is the total number of pediatric bone marrow/stem cell transplants performed at your hospital in the past 12 months? (In the comments provide data source, if data is not available or if no transplants are performed at your hospital) | a)  _________ (number) | N/A |  |
|  |  |  |  | Comments: (e.g., hospital registry, data manager, no data available, no transplants performed at our hospital): |  |  |

**Abbreviations**: BiPAP (Bi-level Positive Airway Pressure); BLS (Basic Life Support); BMT (Bone Marrow Transplant); CAUTI (Catheter Associated Urinary Tract Infections); CLABSI (Central Line Associated Bloodstream Infection); CPAP (Continuous Positive Airway Pressure); HDU (High-Dependency Unit); ICU (Intensive Care Unit); IMCU (Intermediate Medical Care Unit); IV (Intravenous); LR (Lactated Ringer’s Solution); N/A (Not applicable); NICU (Neonatal Intensive Care Unit); NIV (Non-Invasive Ventilation); NS (Normal Saline); PALS (Pediatric Advanced Life Support); PCCM (Pediatric Critical Care Medicine); PHO (Pediatric Hematology-Oncology); PICU (Pediatric Intensive Care Unit); PIV (Peripheral Intravenous); QI (Quality Improvement); VAP (Ventilator Associated Pneumonia); 24/7 (24 hours a day, 7 days a week)

**References:**

1. The World Bank Country Classification. Accessed April 28, 2020. https://datahelpdesk.worldbank.org/knowledgebase/topics/19280-country-classification

2. Friedrich P, Gonzalez M, Lam CG, et al. Development and Validity Testing of PrOFILE: An Assessment Tool for Pediatric Hematology and Oncology Facilities in Low- and Middle-Income Countries. Abstracts from the 51st Congress of the International Society of Paediatric Oncology (SIOP) Lyon, France,. *Pediatr Blood Cancer*. 2019;66:e27989. doi:10.1002/pbc.27989
